# The genome evolution and domestication of tropical fruit mango

**DOI:** 10.1186/s13059-020-01959-8

**Published:** 2020-03-06

**Authors:** Peng Wang, Yingfeng Luo, Jianfeng Huang, Shenghan Gao, Guopeng Zhu, Zhiguo Dang, Jiangtao Gai, Meng Yang, Min Zhu, Huangkai Zhang, Xiuxu Ye, Aiping Gao, Xinyu Tan, Sen Wang, Shuangyang Wu, Edgar B. Cahoon, Beibei Bai, Zhichang Zhao, Qian Li, Junya Wei, Huarui Chen, Ruixiong Luo, Deyong Gong, Kexuan Tang, Bing Zhang, Zhangguang Ni, Guodi Huang, Songnian Hu, Yeyuan Chen

**Affiliations:** 1 Tropical Crops Genetic Resources Institute, Chinese Academy of Tropical Agricultural Sciences & Ministry of Agriculture Key Laboratory of Crop Gene Resources and Germplasm Enhancement in Southern China, No. 4 Xueyuan Road, Haikou, 571100 Hainan China; 2 State Key Laboratory of Microbial Resources, Institute of Microbiology, Chinese Academy of Sciences, 1-3 West Beichen Road, Beijing, 100101 China; 3 CAS Key Laboratory of Genome Sciences and Information, Beijing Institute of Genomics, Chinese Academy of Sciences, Beijing, China; 4 School of Landscape and Horticulture, Hainan University, Haikou, 570208 Hainan China; 5 Center for Plant Science Innovation and Department of Biochemistry, University of Nebraska-Lincoln, Lincoln, NE 68588 USA; 6 Guizhou Subtropical Crops Research Institute, Xingyi, Qianxinan, Guzhou, 562400 China; 7 Joint International Research Laboratory of Metabolic & Developmental Sciences, Key Laboratory of Urban Agriculture (South), Ministry of Agriculture, Plant Biotechnology Research Center, Fudan-SJTU-Nottingham Plant Biotechnology R&D Center, School of Agriculture and Biology, Shanghai Jiao Tong University, Shanghai, 200240 China; 8 Core Genomic Facility and CAS Key Laboratory of Genome Sciences & Information, Beijing Institute of Genomics, Chinese Academy of Sciences, Beijing, China; 9 Institute of Tropical and Subtropical Cash Crops, Yunnan Academy of Agricultural Sciences, Baoshan, 678005 Yunnan China; 10 Guangxi Subtropical Crops Research Institute, Nanning, 530001 Guangxi China; 11 University of Chinese Academy of Sciences, Beijing, China

**Keywords:** Mango genome, Whole-genome duplication, Photosynthesis, Urushiol, Germplasm

## Abstract

**Background:**

Mango is one of the world’s most important tropical fruits. It belongs to the family Anacardiaceae, which includes several other economically important species, notably cashew, sumac and pistachio from other genera. Many species in this family produce family-specific urushiols and related phenols, which can induce contact dermatitis.

**Results:**

We generate a chromosome-scale genome assembly of mango, providing a reference genome for the Anacardiaceae family. Our results indicate the occurrence of a recent whole-genome duplication (WGD) event in mango. Duplicated genes preferentially retained include photosynthetic, photorespiration, and lipid metabolic genes that may have provided adaptive advantages to sharp historical decreases in atmospheric carbon dioxide and global temperatures. A notable example of an extended gene family is the chalcone synthase (CHS) family of genes, and particular genes in this family show universally higher expression in peels than in flesh, likely for the biosynthesis of urushiols and related phenols. Genome resequencing reveals two distinct groups of mango varieties, with commercial varieties clustered with India germplasms and demonstrating allelic admixture, and indigenous varieties from Southeast Asia in the second group. Landraces indigenous in China formed distinct clades, and some showed admixture in genomes.

**Conclusions:**

Analysis of chromosome-scale mango genome sequences reveals photosynthesis and lipid metabolism are preferentially retained after a recent WGD event, and expansion of CHS genes is likely associated with urushiol biosynthesis in mango. Genome resequencing clarifies two groups of mango varieties, discovers allelic admixture in commercial varieties, and shows distinct genetic background of landraces.

## Background

Mango, commonly known as the “king of fruits,” is one of the most popular fruits in the world [1]. Mango is widely cultivated in tropical and warmer subtropical areas in the world. India, China, and Thailand are the top three producers. In 2016, the global production of mango was 46.5 million tons, which ranks as the fifth most produced fruit crop worldwide (http://www.fao.org/faostat/). Mango fruits are mainly consumed fresh, while some are processed into products like nectar, juice, jam, and powder [1]. The fruits demonstrate attractive visual appearance and offer a favorable sensory experience to consumers, making them growingly popular among world consumers. Nevertheless, like many other Anacardiaceae plants such as poison ivy, sumac, and cashew, mango produces phenolic compounds (e.g., urushiols) that can induce contact dermatitis, an undesired quality for fresh mango consumption [2]. The biosynthetic pathways for these compounds remain largely uncharacterized but are believed to arise from initial polyketide synthase-like reactions mediated by chalcone synthase for phenolic ring formation [3].

Mango is the member of the genus *Mangifera* in the Anacardiaceae family [4]. Within this genus, most, if not all, cultivated mangoes belong to the species *Mangifera indica*, although dozens of other *Mangifera* species produce edible fruits [5]. It has a domestication history of over 4000 years within a large area in the Indo-Burmese and Southeast Asia regions but spreads to other parts of the world since the fourteenth century [6, 7]. Traditional varieties have largely been produced through vegetative propagation by grafting of mutated branches, while mango cross-breeding has become dominant since its introduction to the USA, Australia, and China, producing a number of cultivars that have established world popularity [8]. However, considering its long cultivation history and complex genetic backgrounds, it is still largely unknown if there are varieties that can serve as genetic resources different from germplasms currently preserved and produced.

Despite the availability of cytogenetics data [9], transcriptome data [10, 11], and genetic maps [12, 13], whole-genome resources for mangoes are still publically unavailable, which creates difficulties in genomic-based trait improvement and understanding of specialized Anacardiaceae biochemistry underneath. To conquer this, we sequenced and assembled the chromosome-level genome of mangoes. Our analysis reveals that the genome is highly heterozygous, and it has experienced extensive evolution and domestication which may lead to uniqueness and diversity of mango qualities.

## Results

### Genome assembly and annotation

Prior to deep sequencing, a genomic survey of 22 mango varieties with represented genetic background uncovered universally high levels of genome heterozygosity (Additional file 3: Table S1). Among them, the genome of the variety Alphonso, a traditional Indian cultivar, demonstrated a relatively low heterogeneity rate and was thus chosen for whole-genome sequencing and de novo assembly (Additional file 1: Supplementary Notes). The genome was de novo assembled based on single-molecule subreads generated by PacBio Sequel II platform, improved by a combination of paired-end and mate-paired short reads, and incorporated with Hi-C sequencing for scaffolding (Additional file 4: Table S2). The assembled scaffolds were further anchored to genetic maps with 20 linkage groups (pseudomolecules). The final assembly consists of 252 scaffolds, which spans 392.9 Mb in total (Table 1), with the scaffold N50 size of 17.6 Mb and contig N50 size of 3.5 Mb, and with 90.1% anchoring to the linkage groups [12], including 20 pseudomolecules with sizes ranging from 12.2 to 29.4 Mb (Additional file 5: Table S3 and Additional file 1: Supplementary Notes); 391.7 Mb of 392.6 Mb (99.8%) was covered by > 50 PacBio subreads. Ninety-eight percent of the assembled RNA-Seq transcripts were mapped with single scaffolds with aligned length longer than 80% (Additional file 6: Table S4). Of the 6594 genetic markers from published linkage map [12], 6543 (99.2%) were reliably detected in the assembly, and the majority of adjacent markers were located within a short distance in genome sequence assembly (Additional file 2: Figure S1), confirming that the order and orientation of the scaffolds are largely correct. Furthermore, 95.1% of the ultra-conserved core eukaryotic genes based on Core Eukaryotic Genes Mapping Approach (CEGMA) analysis [14] and 95.9% of the single-copy orthologs based on the Benchmarking Universal Single-Copy Orthologs (BUSCO) analysis [15] could be completely detected in the assembly, further confirming the continuity and quality of the assembled genome.

**Table 1:** Statistics for the mango genome assembly and annotation

Assembly and annotation feature	Statistics
Assembly size	392.9 Mb
% of assembly in 20 pseudomolecules	91.1%
Number of scaffolds	252
Scaffold N50 size	17.6 Mb
Number of contigs	420
Contig N50 size	3.5 Mb
CEGMA complete percentage in assembly	95.1%
BUSCO complete percentage in assembly	95.9%
Predicted protein-coding genes	41,251
% of genes in 20 pseudomolecules	90.7%

**Fig. 1:**
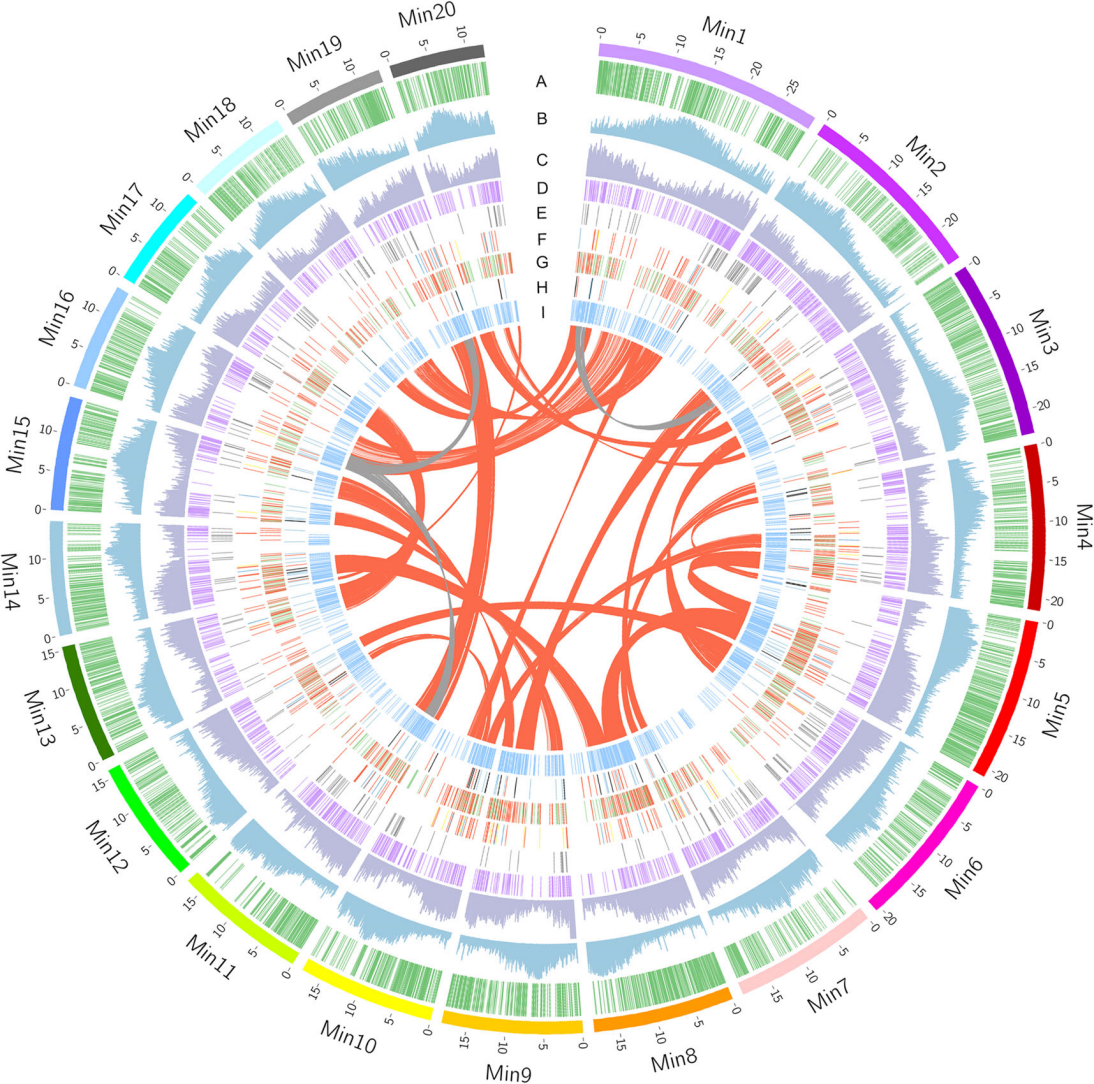
Overview of the mango (*Mangifera indica*) genome assembly. The outer layer of colored blocks is a circular representation of the 20 pseudomolecules, with thick mark labeling each 5 Mb. The distribution of genetic markers mapped to mango chromosomes is shown in (A). Repeat density (B) and gene density (C) are calculated in 100-kb windows sliding in 10-kb steps. Tandem duplicated genes are displayed in (D). Genes involved in disease resistance (E), pigment-related metabolisms (F, yellow lines represent carotenoid synthesis genes, green lines represent chl metabolism genes and red lines represent anthocyanin synthesis genes), lipid metabolism (G, red lines represent genes participant in the synthesis of triacylglycerol, sphingolipid, phospholipid and mitochondrial lipopolysaccharide, phospholipid signaling, and lipid trafficking; the green lines represent the rest lipid metabolism genes) and photosynthesis related genes (H, red lines represent photosystem genes, black lines represent the Calvin cycle genes, and green lines represent genes participant in sucrose and starch synthesis, glycolysis, and Krebs cycle) are also displayed. Transcription factors are shown in (I). The innermost layer shows inter-chromosomal synteny, with the red links representing syntenic blocks retained after a recent WGD in mango genome, and the gray links representing homologs as results of older WGD

Together, we generated a highly contiguous assembly of the heterozygous mango genome. Repetitive sequences account for 40.5% of the mango genome, of which 39.9% was annotated as retrotransposons (Fig. 1; Additional file 7: Table S5). After masking the repetitive sequences, we carried out a combination of de novo gene prediction, homology comparison, and transcriptome-based annotation, as well as quality control to annotate coding genes on the genome (Additional file 1: Supplementary Notes). In total, 41,251 protein-coding genes were annotated (Additional file 8: Table S6, Additional file 9: Table S7, and Additional file 2: Figure S2), of which 37,424 (90.7%) could be located onto the 20 pseudomolecules. In addition, a total of 599 tRNAs, 459 microRNAs, 560 small nuclear RNAs, and 275 ribosomal RNAs were identified in the mango genome.

### Genome evolution

**Fig. 2:**
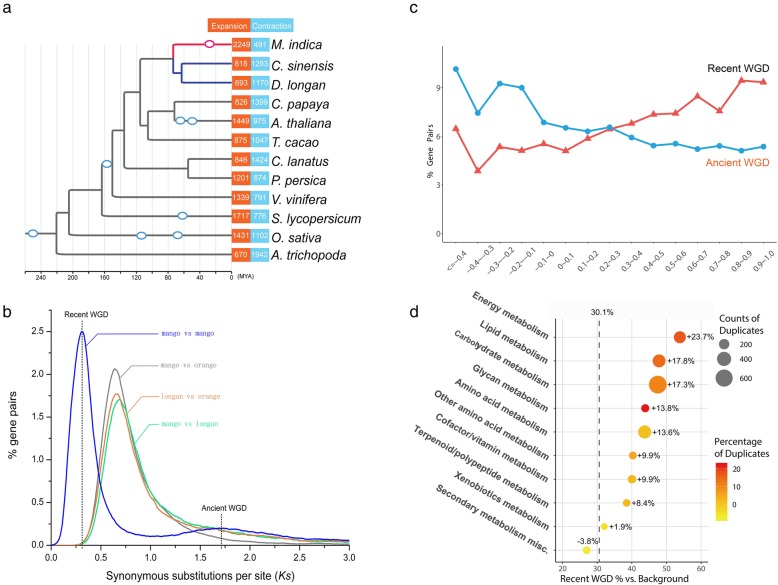
A recent whole genome duplication and resulted gene fate in mango genome. (**a**) Inferred phylogenetic tree across 12 plant species including mango. Estimated WGD events wereindicated with circles. (**b**) Frequency distributions of synonymous substitution rates (Ks) betweenhomologous gene pairs in syntenic blocks of mango-mango, mango-orange, mango-longan andorange-longan. (**c**) Distribution of expressional correlation coefficient of syntenic homologousgene pairs retained from recent WGD and ancient WGD in mango genome. (**d**) Enrichment ofmetabolic genes retained after the recent WGD of mango genome. The vertical dashed linerepresents average percentage of genes retained after the recent WGD

To investigate the evolutionary history of mango genome, we performed a gene family clustering using mango and 11 other representative angiosperm species, including 2 species in the same order Sapindales (*Citrus sinensis* [16] (sweet orange) and *Dimocarpus longan* [17] (longan)), 7 in the same Eudicot clade (*Carica papaya* [18], *Arabidopsis thaliana* [19], *Theobroma cacao* [20], *Citrullus lanatus* [21], *Prunus persica* [22], *Vitis vinifera* [23], and *Solanum lycopersicum* [24]), and 2 outgroup species (*Oryza sativa* [25] and *Amborella trichopoda* [26]) (Additional file 10: Table S8). From the result, 248 single-copy families were used for phylogenetic tree construction and species divergence time estimation, which placed mango as a sister species in adjacent with sweet orange and longan, which is consistent with published results [27] (Fig. 2a). We estimated that mangoes diverged from the ancestor of longan and sweet orange ~ 70 million years ago (MYA).

**Fig. 3:**
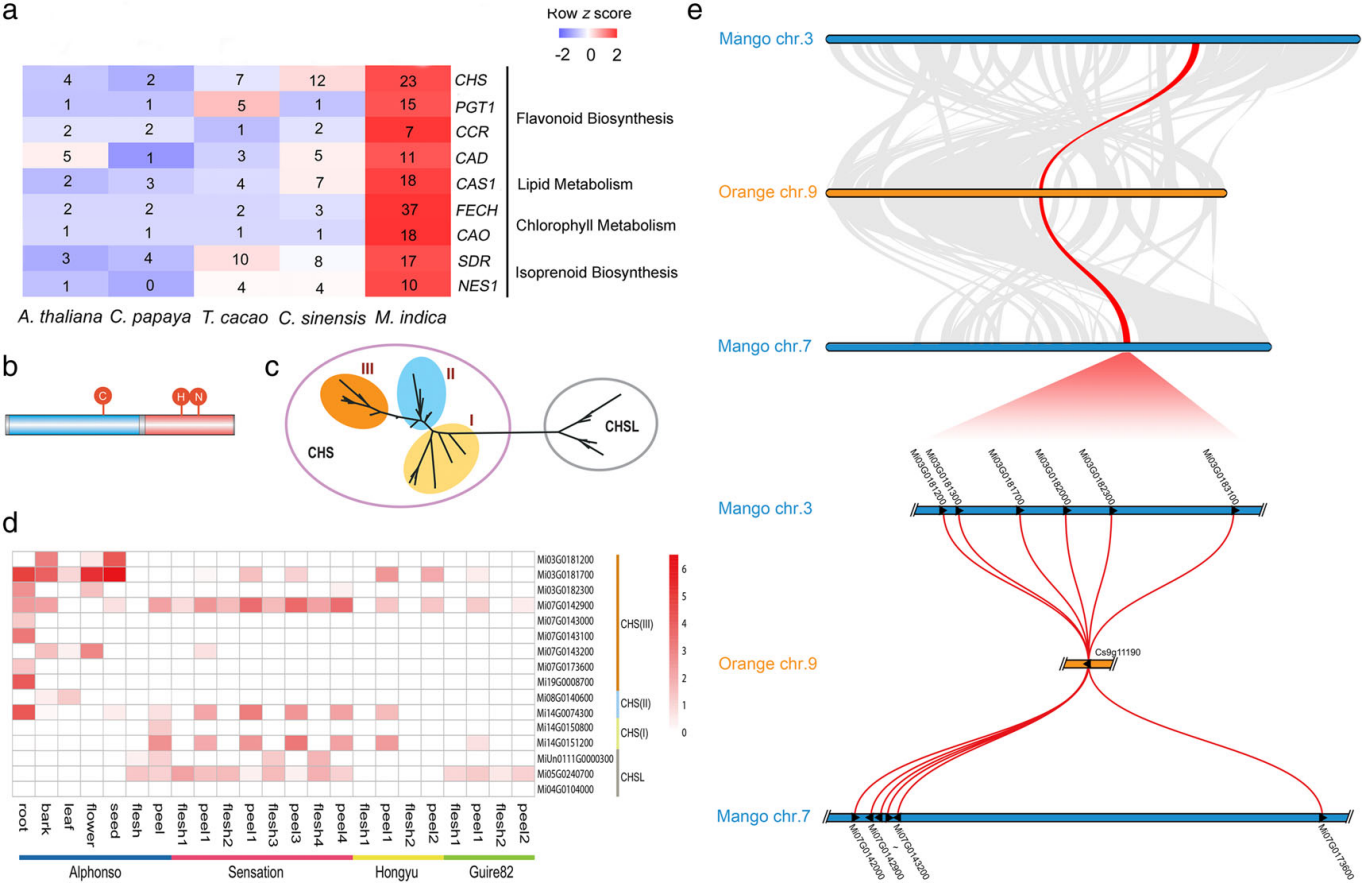
Expansion of genes in metabolism of flavonoids, lipids, chlorophylls and terpenoids. (**a**) Gene family analysis showing expansion of genes in flavonoid biosynthesis, lipid metabolism, chlorophyll metabolism and isoprenoid biosynthesis, including CHS family. Numbers showing in cells represent genes counting of each family in each species. (**b**) Gene structure of a typical CHS peptide (here shows mango gene Mi07g07250). The blue and red blocks represent two conserved domains PF00195.15 and PF02797.11, respectively, and the red circles suggest three conserved residues, Cysteine, Histidine and Asparagine, respectively, within the sequence. (**c**) Topology of phylogenetic tree of genes containing conserved domains PF00195.15 and PF02797.11 from mango, sweet orange and Arabidopsis. CHS-encoding genes are classified in a distinctive class, here named CHS. (**d**) The expression pattern of *CHS* genes in mango cultivars. Unexpressed genes were not showed in this figure. (**e**) Macrosynteny and microsynteny among mango chr.3, orange chr. 9 and mango chr. 7. Top: macrosynteny patterns among mango chr.3, orange chr. 9 and mango chr. 7. Co-synteny regions are linked with grey belts, except synteny of the regions harboring *CHS* genes, which are highlighted with a red belt. Bottom: microsynteny of the regions harboring CHS genes among the three chr. The CHS genes are represented by triangles, and are linked with red lines

To detect whole-genome duplications (WGD) in mangoes, we performed a genomic synteny analysis based on the self-comparison of mango coding genes, which identified 41 large collinear blocks with at least 50 homologous gene pairs. Among them, 38 collinear blocks have similar (0.3–0.4) median synonymous substitutions values (*Ks*) for homologous gene pairs (Additional file 11: Table S9). These collinear blocks were distributed across the 20 pseudochromosomes, spanning 50.7% length of chromosomes (181.3 Mb/357.7 Mb) and covering 62.1% of protein-coding genes (23,260/37,424) (Additional file 12: Table S10), which strongly supports a recent WGD event in mango genome. An exemplary illustration of co-synteny is shown between chr. 3 and 7 (Fig. 3e). In contrast to the findings of biased fractionation following WGD in maize [28] or *Arabidopsis* [29], there was no apparent difference for region lengths or gene density between the mango collinear blocks (Additional file 11: Table S9), indicating that the mango-specific WGD might be an auto-diploidization event. The calculation of *Ks* for mango vs. orange, mango vs. longan, and orange vs. longan collinear orthologs indicated that the WGD event occurred after the split of mango lineage and the ancestor of orange and longan (Fig. 2b); no WGD event was detected after the split in either genome of sweet orange and longan (Additional file 2: Figure S3). This WGD event in the mango genome might date back to ~ 33 MYA by mapping the WGD event onto the phylogeny.

Within the 5369 homologous gene pairs retained post-WGD, 508 gene pairs are transcriptional factors, accounting for 9.6% of the retained genes, more than the average level in the genome (5.6%, 2342/41,251). Consistently, Gene Ontology analysis revealed the gene pairs retained post-WGD are enriched in the “DNA binding” category of molecular function (GO:0003677, DNA binding, *p* = 4.42e−13) (Additional file 13: Table S11). These results suggest the preferred retaining of essential and regulatory genes after WGD in the mango genome, consistent with the dosage hypothesis that genes with large number partners are preferred to be retained after WGD [30]. In addition, the expressional similarities analysis on multiple tissues found that the gene pairs retained from recent WGD event were significantly greater than those retained from ancient WGD (Additional file 14: Table S12 and Fig. 2c). The omega values (*Ka*/*Ks*) for most of collinear homologous gene pairs were smaller than 1 (Additional file 2: Figure S4), indicating that protein neofunctionalization may not be the predominant status for the retained genes from recent WGD.

Mango-specific recent WGD may also impact the different metabolism categories with distinct contributions. For instance, the percentage of retained gene from recent WGD in energy metabolism, glycan biosynthesis and metabolism, carbohydrate metabolism, and lipid metabolism was about 48.4–53.8%, much greater than the genome average 30.1% (Fig. 2d). Notably, genes involved in photosynthesis were preferentially retained after recent WGD (53.6%), especially for genes that participated in the Calvin cycle (56.1%) (Additional file 15: Table S13). Half of the sugar metabolism-related genes were the results of recent WGD, and the majority of pathway members in sucrose synthesis (7/10) and starch synthesis (8/9) have recent WGD-retained duplicated genes (Additional file 16: Table S14). These findings indicated recent WGD-retained genes may preferentially take participant in the assimilation, storage, and utilization of CO_2_. Lipid genes were also found to be preferentially retained post-WGD (47.9% vs. 30.1% in general). Interestingly, genes of lipid-saccharide conjugate biosynthesis were preferentially retained, including genes in galactolipid, sphingolipid, and liposaccharide metabolisms. In addition, genes related to lipid trafficking and phospholipid and triacylglycerol biosynthesis were preferentially retained in the mango genome (Additional file 17: Table S15). Isoprenoid synthesis genes are also preferentially retained, especially for the genes involved in synthesis of precursor isopentenyl diphosphates (IPP), that is, most of the genes in the cytosolic mevalonate (MVA) pathway (4/6) and plastidic 2-*C*-methyl-d-erythritol-4-phosphate (MEP) pathway (6/7) have duplicated copies retained from recent WGD (Additional file 18: Table S16).

### Gene expansion

Gene family analysis among 12 plant species provided hints for a better understanding of the genetic basis of mango biology. Among the 3791 gene families with at least 20 members across 12 plants, we detected expansion of 2249 gene families and contraction of 491 gene families in the mango genome (Fig. 2a and Additional file 19: Table S17). High ratio of gene expansion to contraction (4.5×) in the mango genome is in striking contrast to sweet orange (0.6) and longan (0.4). Among the 12 plants we investigated, other species with recent WGD events (< 80 MYA), including *Arabidopsis*, rice, and tomato, also demonstrated higher expansion/contraction ratio than other species, but the highest ratio was in mango genome, reflecting a relatively recent occurrence of the WGD event in the mango genome. Gene Ontology (GO) enrichment analysis showed that the significantly expanded gene families in mangoes are enriched in basic biological functions, primarily in phosphorylation process and nucleotide-binding function; also enriched are genes in response to biotic stimulus response (*p* < 1e−15) (Additional file 20: Table S18).

Considering that mango individuals suffer from diseases through all stages of their life cycle that result in rot, necrosis, spots, etc., we analyzed the gene composition and phylogeny of disease resistance-related gene families in the mango genome and compared them to the phylogenetic close plants. In total, we identified 437 genes encoding nucleotide-binding site and leucine-rich repeat (NBS-LRR), as well as 296 receptor-like genes and 13 genes of lipoxygenase. Among these gene families, receptor-like genes, NBS-LRR-encoded genes experienced expansion in mango genome compared to sweet orange genome (Additional file 21: Table S19).

### Expansion of CHS genes: relation to specialized phenolic biochemistry

Among the expanded families, several mango gene families are involved in flavonoid biosynthesis, lipid metabolism, chlorophyll biosynthesis, and isoprenoid synthesis (Fig. 3a). Notable among this expansion is chalcone synthase gene (CHS) family. Our results show that the mango CHS gene family is composed of 23 members in the mango genome, whereas only 12 and 4 were identified in sweet orange and *Arabidopsis*, respectively (Fig. 3a). The CHS family members were confirmed by the identification of the genes containing conserved domains PF00195.18 and PF02797.14 (Fig. 3b). Phylogenetic analysis showed that the genes in CHS family split into 2 independent groups, one of which clustered the majority of the mango CHS family members (19 of 23) together with a *Arabidopsis* gene that has been demonstrated to be a bona fide CHS gene [31]; the remaining 4 mango genes were clustered with *Arabidopsis* genes encoding CHS-like (CHSL) hydroxyalkylα-pyrone synthases [32] (Fig. 3c). Based on transcriptomic analyses, the expression of *CHS* genes were generally higher than that of CHSL members in mangoes (Fig. 3d).

**Fig. 4:**
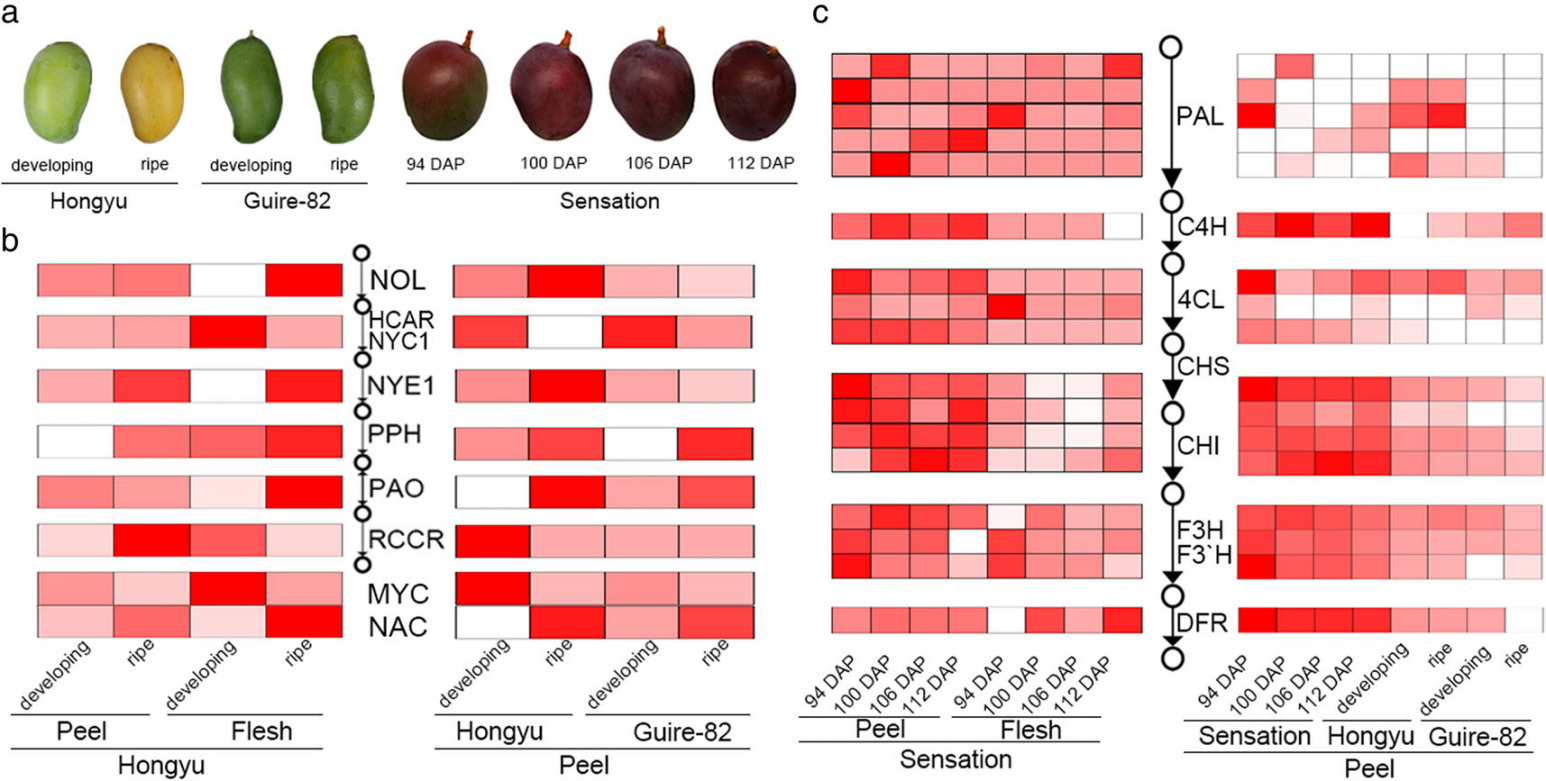
Expression of genes in chlorophyll degradation and anthocyanin biosynthesis in mango fruits. (**a**) Demonstration of peel colors of three representative mango varieties. Peel of developing fruit of Hongyu is green, while that of ripe fruit is yellow; peel of Guire-82 keeps green even when ripen; peel of Sensation demonstrates red color. (**b**) Expression of genes in chlorophyll degradation pathway in peel and flesh of Hongyu fruits as well as peel of Guire-82. (**c**) Expression of genes in anthocyanin biosynthesis in peel and flesh of Sensation fruits, as well as peels of Hongyu and Guire-82

Mango produces urushiols and related anacardic acids, characterized by an aromatic ring linked to a hydrocarbon tail of differing chain lengths and unsaturation [2]. Urushiols are characteristic of many Anacardiaceae species like poison ivy, sumac, cashew, and mango. Current knowledge is limited on the mechanisms of urushiol biosynthesis, but the first committed step in the pathway is believed to be catalyzed by CHS [3]. CHS also generates the aromatic ring of anthocyanins, the red fruit peel pigment found in many consumer-preferred mango varieties (e.g., Sensation and Alphonso) [33] (Fig. 4a and Additional file 2: Figure S5). Expansion of *CHS* genes in mango genome highlights its important roles in the evolution of the Anacardiaceae clade. To shed light on roles of the *CHS* group in mangoes, we further analyzed the sequence, phylogeny, expression, and synteny characteristics of these genes in the mango genome.

Phylogeny further split the CHS group genes into three clades, namely clades I, II, and III (Fig. 3c, Additional file 2: Figure S6, Figure S7 and Figure S8). Three and two CHS genes are harbored in clades I and II, respectively. Only Mi14G0074300 and Mi14G0151200 had evident expressions, both showing remarkably higher expression in peel than in flesh in the red-peel varieties Alphonso and Sensation, while not significant expression was observed in mature fruits of yellow-peel variety Hongyu and green-peel variety Guire 82 (Fig. 3d). Considering the red color of the mango peels is attributed to the accumulation of anthocyanins, the expression pattern suggests the role of CHSs in clades I and II in anthocyanin biosynthesis in mangoes. A majority of *CHS* genes (14 of 19) are in clade III, in which only one sweet orange homolog and one *Arabidopsis* homolog are present, indicating that the extensive expansion of the *CHS* genes mainly occurred among the clade III members in mangoes. We asked if the expansion is a widely occurring event in Anacardiaceae, and retrieved *CHS* homologs from genomes of pistachio (*Pistacia vera*) and *Sclerocarya birrea*, and those from assembled contigs of publically available transcriptomes of poison ivy (*Toxicodendron radicans*) and sumac (*Rhus chinensis*), two other Anacardiaceae species producing urushiols (Additional file 3: Table S20) [34, 35]. Phylogeny clearly showed the contigs together with mango CHSs are clustered into three clades, and the majority of contigs are grouped with mango clade III genes, in agreement with mango peptide phylogeny (Additional file 2: Figure S7 and Figure S8). The results suggested the extensive expansion of CHS was a commonly occurring event in Anacardiaceae, which was originated from a single *CHS* copy.

Many clade III genes (12 of 14) are located in two syntenic clusters in the mango genome, a result of the recent WGD, which is syntenic to a region in Chr. 9 of sweet orange genome with a single *CHS* gene (Cs9g11190), suggesting the WGD medicated gene expansion occurred after the split of Acanardiaceae and Rutaceae (Fig. 3e). Besides the regions harboring CHS genes, the co-synteny can be found among the majority of the chr. 3 and chr. 7 of mango and chr. 9 of sweet orange (Fig. 3e). Each cluster has several *CHS* genes, which are likely results from tandem duplications (Fig. 3e). Gene structure and sequence identity revealed that Mi03g0182300 and Mi07g0142900 show higher similarity with the sweet orange *CHS* gene (Cs9g11190), likely serving as ancestral genes of the tandem duplications (Additional file 24: Table S22). All the three sites key to the CHS activity are conserved among the mango CHS peptides (Additional file 2: Figure S9).

Despite extensive duplications, class III *CHS* genes in early stages of tandem duplication tended to express highly compared to the products of later duplications (Fig. 3d). Only two genes showed evident expressions in fruits, and they exhibited ubiquitously higher expression in peels than in flesh of the varieties with different colors when ripe, indicating that the *CHS* genes in clade III are not involved in anthocyanin biosynthesis in mangoes. Moreover, urushiols are ubiquitously more abundant in peels than in flesh in different mango varieties, as there are abundant resin canals where urushiols are produced [36]. Ubiquitous higher expression of the clade III *CHS* genes in peels in different mango varieties suggest their roles in regulating biosynthesis of urushiols and related phenols in mangoes [37, 38] (Fig. 3d).

### Mango fruit peel pigmentation

During the process of fruit development and ripening, mango fruit peels usually undergo dramatic, mild, or no color changes, resulting in combinations of yellow, red and green pigmentation that is mostly variety-dependent. For example, fruits of the variety Sensation undergo rapid color changes, resulting in light to dark red coloration of peels in the process of fruit development (Fig. 4a). Consistent with the long-term understanding that red color of fruits is a result of anthocyanin accumulation, most genes in the anthocyanin biosynthesis pathways exhibited tremendously higher expression levels in red-peel Sensation than in peels of red pigment-free Hongyu and Guire-82 fruits (Fig. 4b). We also observed remarkably higher expression levels of most anthocyanin biosynthesis genes in Sensation fruit peels than in flesh, consistent with the observations in the leaves that the light stimulated the synthesis of anthocyanin and the accumulated anthocyanin protect plants against the damage of strong light [39, 40] (Fig. 4b).

During the fruit ripening, the fruit peel color of some varieties turns from green to yellow, a process called degreening. This is virtually a process of chlorophyll degradation mediated by Chl catabolic genes (CCGs) [41]. Hongyu is a typical variety whose fruits experience degreening process and exhibit yellow-peel, while Guire82 is a Chinese cultivar derived from yellow-peel Neelum and represents atypical stay-green peel (Fig. 4a). No major differences in the expression of CCGs were detected between the green-peel Guire82 and the yellow-peel Hongyu during ripening (Fig. 4c).

### Mango germplasm diversity

We selected 48 *M. indica* accessions and 4 additional species of *Mangifera* for whole-genome resequencing, including 35 typical cultivars representing the history of mango breeding in different areas of the globe and 13 landraces germplasms collected from remote areas in South China. Across the 52 accessions plus the variety Alphonso, a total of 21.04 million high-confidence variants, or 53.9 variants per kilobyte in average, were identified. They include 19,433,034 SNPs, 635,320 insertions, and 972,376 deletions; 4,297,808 variants (20.4%) were located in genic regions, including 542,626 synonymous, 828,252 nonsynonymous, and 2,849,557 intronic variants. Among them, 69.1% have a minor allele frequency of less than 10%. This tendency was even greater for functional variants, as the frequencies of nonsense mutation sites were 0.15% with MAF < 10%, while its value was decreased to 0.03% with MAF > 90% (Additional file 25: Table S23).

**Fig. 5:**
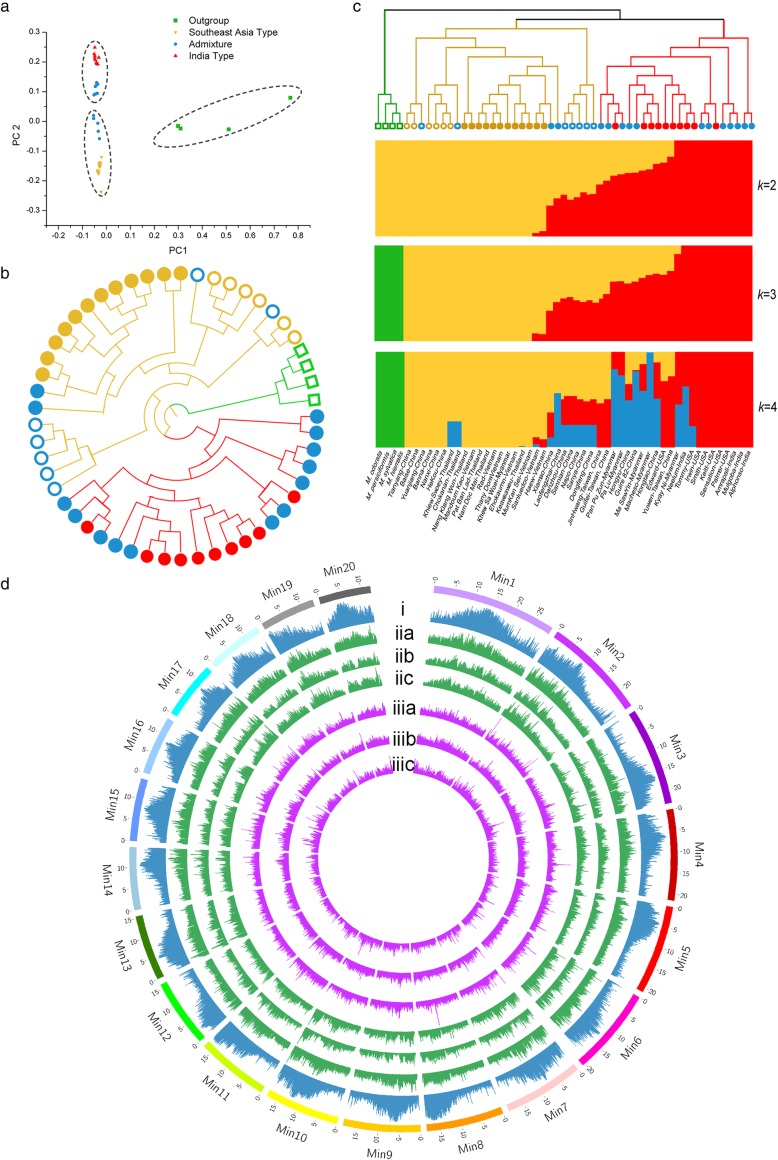
Genomic diversity of *M. indica* varieties and relatives within *Mangifera*. 49 *M. indica* germplasms and 4 otherspecies in the genus of *Mangifera* were sampled for the analyses. (**a**) PCA analysis of the samples using SNP markers. The three groups indicated by phylogenetic (**b**) and STRUCTURE (*k*=3) (**c**) analyses were circled, respectively. Germplasms with different backgrounds are represented with dots with different shapes and colors. (**b**) Neighbor-joining phylogenetic tree of the samples in polar layout based on SNPs. Clades of the three major groups are indicated with different colors as indicated in STRUCTURE analysis (*k*=3). Tips of outgroup germplasms are indicated with green hollow blocks. Tips of traditional and commercial varieties are labeled with filled rounds, and those representing landraces are suggested as hollow circular forms. Traditional varieties and commercial cultivars without admixture are labeled in red, and germplasms representing Southeast Asia varieties and landraces are in yellow, while those with allele admixture are in blue. (**c**) STRUCTURE analysis of the samples, with each color representing one population, and the length of each color segment in each vertical bar representing the proportion contributed by ancestral populations. On the right is assumed number of clusters (*k*), and below is the name or origin of the samples. (**d**) Circos demonstration of genetic diversity. Outer circle represents 20 pseudo-molecules of mango genome. i, contents of repetitive elements; iia- iic, nucleotide diversity (π) of other *Mangifera* species, varieties of Southeast Asia and India germplasms, respectively. iiia-iiic, population differentiation (*F*_ST_) levels of other *Mangifera* species vs. germplasms of Southeast Asia, other *Mangifera* species vs. India germplasms, and those of Southeast Asia vs. India, respectively

As expected, all the mango varieties form a group distinct from the four other *Mangifera* species (Fig. 5a, b). Phylogenetic inference splits the mango varieties into two distinct groups, with indigenous varieties from Southeast Asia residing in one group and traditional varieties from India in the other. This coincides with the long-proposed two centers of domestication, one in India and the other in continental Southeast Asia [42, 43] (Fig. 5b). The overall *F*_ST_ between *Mangifera* wild relatives and Southeast Asia/India varieties (0.1747/0.1856) is higher than that between Southeast Asia and India varieties (0.1358). Furthermore, the USA varieties are more closely related to India varieties than to Southeast Asia varieties, agreeing with previous analysis using 25 microsatellite loci [42] (Fig. 5b, c). Landraces collected in South China were clustered together with South East germplasms (Fig. 5b), some of which experienced allelic admixture (*k* = 3, 4; Fig. 5c). Although grouped with India germplasms, most of the commercial varieties experienced allelic admixture (Fig. 5c). India and Southeast Asia varieties have a comparable level of genetic diversity (*π*) (0.0084 and 0.0094) as that of other *Mangifera* species (0.0094). The regions of high *π* values in the mango genome coincide with highly repetitive areas (i, iia–iic; Fig. 5d). No significantly enriched functional roles were observed in genetic diversity-declined regions compared between *Mangifera* wild relatives and India type/Southeast Asia type with different parameters.

Despite the widespread admixture, inbreeding played important roles in the mango breeding history. The values of the inbreeding coefficient and cumulative length of runs of homozygosity (ROH) varied within different cultivars (Additional file 26: Table S24 and Additional file 2: Figure S10). As expected, the admixture of both the Southeast Asia and India germplasms have lower inbreeding coefficient and small amounts of ROH. Interestingly, we found the length of ROH was related to genomic heterozygous rates in mango cultivars (Additional file 2: Figure S11). The Southeast Asia germplasms have a higher inbreeding coefficient and have more regions of runs of homozygosity (ROH). Three India and USA germplasms close to the variety Alphonso have the largest inbreeding coefficient. The excess of ROH by inbreeding is the reason why Alphonso have a lower level of heterozygosity. The USA cultivar Irwin also has larger regions of ROH, suggesting the existence of inbreeding in Irwin breeding.

## Discussion

A high-quality reference genome for mangoes can facilitate mango molecular breeding and evolutionary studies of Anacardiaceae. The mango genome is highly heterozygous and also experienced a recent WGD event, rendering substantial challenges for mango genome assembly [44]. Sequencing technologies producing long reads have facilitated the recent assembly of highly heterozygous genomes, such as those of durian [45], oak [46], and *Gnetum montanum* [47]. We addressed the issues of high heterozygosity and WGD primarily by using long (PacBio) reads, in association with NGS sequencing, Hi-C mapping, and a high-density integrated linkage map, through which we have produced a chromosome-level genome assembly for mango (*Mangifera indica*). To our knowledge, this is the first and most complete genome assembly of mango. The genome assembly provides a useful resource for mango breeding and also is valuable for understanding the biochemistry and evolution of specialized metabolism for urushiols and related phenols in Anacardiaceae.

WGD events, or polyploidization, occurred throughout the routes of plant evolution, which are important drivers for specialization and emergence of novel traits and functions [48–51]. Consequences of WGD offer genetic preconditions for successful domestication which is responsible for the advent of many crops [52]. The recent WGD in mango genome (~ 33 MYA) occurred long after the split of Anacardiaceae from Rutaceae and Sapindaceae (~ 70 MYA based on our results) and even after the advent of the family Anacardiaceae (54.8–74.9 MYA) [53, 54]. Mango belongs to the genus *Mangifera* which can be classified under the tribe Anacardieae. Despite the availability of molecular systematics data of Anacardiaceae [55], a detailed molecular clock dating of the species within the family is lacking, and no other chromosome-level Anacardiaceae genome is currently available [56], which hamper our detailed estimation of WGD event time. However, we observed preferential post-WGD retaining of duplicated genes involved in energy metabolism, such as photosynthesis and lipid biosynthesis. Considering that the recent WGD event occurred within the period of drastic decline of atmospheric carbon dioxide concentrations resulting in a descent into icehouse climate [57, 58], it is appealing to hypothesize that the preferential retaining of the duplicated energy metabolism genes is the result of adaptive evolution to cope with the decline of carbon dioxide as an essential substrate for carbon fixation and shrinking capability of the plant photosynthesis and energy assimilation by icehouse climate.

Urushiols and related phenols (e.g., anacardic acids) are produced in many Anacardiaceae plants, with a 15- or 17-carbon side chain that is responsible for allergic skin rash [36]. These phenols are likely defensive molecules against fungi, insects, and herbivorous vertebrates [59–61]. Nevertheless, they have the potential for the treatment of cancer and skin and viral diseases [62]. Phenolic compounds are diverse, and the diversification of phenols is based on the general phenolic biosynthesis pathway, in which early genes like chaconne synthase (CHS) have important contributions to the metabolic flow of phenols in plants [31]. Extensive expansion of CHS genes in the mango genome highlights the important roles of CHS in mango. The expansion before the recent WGD but after the split of Anacardiaceae and ancestor of Rutaceae and Sapindaceae indicates the *CHS* genes might have been expanded in the early stages of Anacardiaceae emergence. Our results show that extensive duplications lead to a large number of members in clade III of *CHS* genes, and, for the highly expressed genes, they tend to be highly expressed in barks and peels rather than in the flesh of all the four varieties we investigated; this is comparable to high accumulation of urushiols in these tissues in mangoes, suggesting their important roles in the biosynthesis of urushiols in mangoes [2, 63, 64]. As urushiols are specific to and commonly accumulated in Anacardiaceae to varying levels, it will be interesting to investigate if *CHS* genes follow similar patterns of gene family evolution in other species when more Anacardiaceae genomes are available which might help understand the mechanisms and evolution of the family-specific phenol biosynthesis.

It is widely accepted that there are two centers of domestication: one in India, a majority of which are monoembryoonic, and the other in continental Southeast Asia, most of which are polyembryonic [42, 43]. Mango cultivars in Florida, USA, are unique, as they have a long history of breeding, and many of the cultivars are descendants of Mulgoba, the only one survived among the six grafted mangoes introduced from India in the late nineteenth century [8]. During the past decades, Chinese breeders have successfully bred several varieties which demonstrate superior performance. With the availability of high-quality reference genome and resequencing data, we have an unprecedented opportunity to closely study genetic components and compare mango germplasms with different backgrounds. This can help clarify relationships of many mango varieties, on which efforts have been made by other approaches [8, 42]. Our results show that the mango varieties can be clustered into two groups, coinciding with the proposed two centers of domestication, and allelic admixture was observed in the genomes of commercial varieties.

Although grouping with Southeast Asia germplasms, landraces indigenous in China forming distinct clades and some have clear allelic admixture. These represent unique genetic resources for future mango breeding endeavors. Landraces have long been recognized as a source of traits for improving yield, nutrition, and abiotic stress adaptation. They are especially important as agricultural production is affected by worldwide climate change [65]. Varieties widely cultivated are facing common issues such as alternate bearing, narrow ripening window, poor fruit quality, and cold sensitivity [2]. It is essential to broaden the repository of genetic resources by exploring more exotic germplasms. The landraces in South China represent a valuable resource for future breeding. However, the agronomic and quality characters of these landraces are currently poorly understood, and the genetic resources are threatened by modern agricultural practices and expansion of industries. It is urgent to proceed with comprehensive studies on these landraces. We believe these landraces might not be only germplasms distinct from the mainstream varieties. The availability of the mango genome lays the ground for a more thorough survey of landraces, which would be critical for the improvement of agronomic and quality traits of current mango varieties.

## Conclusions

We have generated a mango genome assembly, which is, to our knowledge, the first publically available genome resource for mangoes. This provides crucial information for the study of the evolution of not only mangoes, but the Anacardiaceae family. And this will facilitate the establishment of genome-enabled breeding programs for mango. We estimated that the mango genome underwent an event of whole-genome duplication (WGD) about 33 million years ago. Interestingly, duplicated genes involved in photosynthesis and lipid metabolism are preferentially retained in the mango genome, which likely provides adaptive advantages to sharp historical decreases of concentrations of atmospheric carbon dioxide. Sixty-eight percent of gene families were expanded in the mango genome; among them, genes of *chalcone synthase* (*CHS*) were extensively duplicated, which are mostly results of tandem duplications prior to WGD. Particular *CHS* genes showed universally higher expression in peels among mango varieties, which are likely involved in the biosynthesis of urushiols and related phenols, a group of Anacardiaceae-specific phenols which can induce contact dermatitis. Two distinct groups of mango varieties through genome resequencing, with commercial varieties clustered with India germplasms, which demonstrate allelic admixture. Although grouped with Southeast Asia germplasms, landraces indigenous in South China formed distinct clades, some of which showed admixture.

## Methods

### Genome survey sequencing

Genome survey sequencing was firstly carried out to identify mango accessions suited for whole-genome sequencing. Twenty-two mango cultivar/landrace samplings from the Chinese Academy of Agricultural Sciences Tropical Resources Institute of tropical crop varieties (Hainan, China) were sequenced with Hiseq2000 at about 20× genomic coverage. Leaf genomic DNA isolation, paired-end library construction, and sequencing were carried out as described in our previous publication [66]. Genome size, heterozygosity, and repeat content were estimated with a *k*-mer method using Jellyfish (v2.1.3) [67]. Of the 22 sequenced mango cultivars/landraces, Alphonso has a relatively smaller genome size, lower heterozygosity rate, and repeat content. Considering that Alphonso has a clear breeding history and was used as primary breeding germplasm, we chose it as the material for the whole-genome short-gun sequencing using a single molecular sequencing strategy. Meanwhile, GenomeScope [68] and ALLPATHS-LG (v52488) [69] were also used to investigate the genome profile of Alphonso cultivar. The estimated genome size of Alphonso cultivar is ~ 360 Mb, and the estimated heterozygosity rate is ~ 1.5%. The raw reads of genome survey sequencing are deposited in the NCBI Sequence Read Archive under project ID PRJNA487154 [70]. For additional information, see Additional file 1: Supplementary Notes.

### Transcriptome data production and analysis

Total RNA was extracted from mango tissues using TRIzol. Subsequent mRNA extraction and mRNA-seq libraries were conducted using Kapa transcriptome kits and sequenced with Hiseq3000. Qualified reads ware mapped to mango assembly guided by gene annotation models using hisat2 (v2.0.4), and the expression level for each gene was performed by Stringtie (v 1.2.3) [71]. Pearson correlation coefficient for each gene pair was calculated with custom PERL scripts. The raw data of transcriptomic sequencing are deposited in the NCBI Sequence Read Archive under project ID PRJNA487154 [70].

### Genome sequencing and assembly

The single-molecule long reads were generated from 1 cell run on the PacBio Sequel II Platform. A total of 86.5-Gb long reads (~ 240× based on estimated 36) were generated and de novo assembled using CANU (version 1.8) [72]. The pair-end and mate-pair short reads were generated by HiSeq2000 and MiSeq platform, including 2 TruSeq PCR-free pair-end libraries with an insert size of 180 bp and 500 bp and 4 Nextera mate-pair libraries with an insert size of 3 kb, 5 kb, 8 kb, and 10 kb. The short reads were also independently assembled by ALLPATHS-LG (v52488) [69] to investigate the genome profile including estimated genome size and SNP rate for cross-checking. The initial Canu assembly was corrected using a combination of long and short reads with Pilon (v1.23) [73]. Duplicated assembled haploid contigs were purged using PurgeHaplotigs [74], which reduced the assembly from 624.85 to 363.08 Mb. A Hi-C library was constructed and sequenced on the Illumina NovaSeq platform for chromosome-level scaffolding. With the Hi-C library, the purged contigs were anchored into super-scaffolds using Juicer [75] and 3d-dna pipeline [76]. ALLMAPS (version 1.0) [77] was used to anchor the Hi-C super-scaffolds with unique mapped genetic markers from the previous published mango genetic map [78]. Finally, the genome assembly contains 20 pseudochromosomal molecules, 2 organelles, and 230 unplaced scaffolds. The raw data of genome deep sequencing (second and third generation sequencing) together with genome assembly are deposited in the NCBI Sequence Read Archive under project ID PRJNA487154 [70]. For additional details about the genome sequencing and de novo assembly, see Additional file 1: Supplementary Note.

#### Genomic assembly quality evaluation

To assess the quality of the assembly, we mapped all the pair-end and long reads to the assembly for their mapping rate, which indicates the quality and integrity of the assembly. All the pair-end reads were mapped using BWA-MEM (v0.7.15) [79], and all the PacBio long reads were mapped using minimap2 [80]. the integrity of the protein-coding genes of the assembly was evaluated using Benchmarking Universal Single-copy Orthologs (BUSCO) analysis (v3.0.2, embryophyta_odb9) [15] and Core Eukaryotic Genes Mapping Approach (CEGMA) analysis (v2.2) [14]. Genome completeness was further evaluated by the mapping of 6594 mango genetic markers [12] and 20,920 Trinity (v 2.2.0) [81] assembled transcripts (length > =1 kb) from RNA-seq data using BLAT software (v34x10, [82]).

#### Genome annotation

In prior to gene prediction and annotation, the library of repetitive sequences was ab initio constructed using RepeatModeler. By using this library, repetitive sequences were annotated, classified, and soft-masked by RepeatMasker (http://www.repeatmasker.org/). Transcripts were constructed using a combination of HISAT2 [83], Stringtie [71], and TACO [84]. The ORFs on the transcripts were extracted using TransDecoder within the PASA pipeline [85]. The homologous from the Uniprot database (taxonomy: 3398 [Magnoliophyta]) were mapped to the genome using GenomeThreader [86]. The ab initio prediction of protein-coding gene was carried out by the BRAKER2 pipeline [87]. The results from ab initio prediction, homologs, and transcription evidences were integrated using EVM software (v2012-06-25) [88] and further curated by removing frame-shifts and redundancies using the gffread tool from Cufflinks [89]. Meanwhile, retro-transposon (RT) genes were identified by HMMER [90] with the Pfam database and removed from the final annotation. Transcription factor identification was conducted in the plant TF database (http://planttfdb.cbi.pku.edu.cn/prediction.php) [91]. Non-protein coding genes were detected by the homologous searching of the Rfam database [92] using Infernal (v1.1.2) [93]. Protein annotations were carried out by searching NCBI non-redundant protein database, InterPro [94], and KEGG [95] databases. GO information was extracted from InterPro annotation. For additional details about the genome annotation, see Additional file 1: Supplementary Notes. The GO enrichment analysis for the retained duplicated genes was performed by GOEAST [96] (http://omicslab.genetics.ac.cn/GOEAST/).

#### Comparative genome analysis

Twelve species (including mango) representing the major plant domains were selected for phylogenetic analysis. All-versus-all BLASTP [97] searching results (*e* value threshold 1e−5) were used for gene family construction using OrthoMCL (v2.0.9) [98] with series *I* values. The nucleotide sequences of 248 single-copy genes were concatenated from CDS alignments guided by individual protein alignments using Clustalw2 (v2.1) [99] The best model (HKY+I+G) was selected by Jmodeltest (v2.1.7), and then a ML tree was constructed with 100 bootstrap value using PhyML (v3.1) [100]. And the resulting ML tree was used as an input tree for the Café software and PAML MCMCTree program. The PAML [101] MCMCTree program was used to estimate the species divergence times with the HKY85 model. We used the *A. thaliana* and *C. papaya* divergence time (68–72 million years ago) [18] and the monocot and eudicot divergence time (130–240 million years ago) [23] as calibrators. The MCMC analysis was run for 20,000 generations, using a burn-in of 2000 iterations. Café software (v3.2, [102]) was used to identify the gene family that had undergone expansions or contractions for the 2903 gene families with at least 20 members among 12 plant genomes.

#### Genome duplication analysis

MCScanX [103] was used for syntenic region detection with the all-to-all BLASTP results (blocks with at least 10 pairs homologous genes and the gap was less than 5 genes) for orange/mango, orange/longan, mango/longan, and mango/mango. The *Ks* between the syntenic homologous gene pairs was calculated by PAML (v4.8) [101] using the YN00 NG model.

For the definition of mango duplicated gene, firstly, the duplicate_gene_classifier module in MCScan software was used to classify the duplicate genes into WGD/segmental duplication (≥ 10 homologous gene pairs in collinear blocks), tandem (consecutive repeat), proximal (in nearby genomic region but adjacent within 10 genes), or dispersed (other modes than segmental, tandem and proximal) duplications. The remaining genes were defined as singletons. Secondly, the WGD/segmental duplicated genes were further classified into two subclasses: the gene pairs located in syntenic blocks with the median *Ks* of 0.3–0.4 were defined as the genes retained from recent WGD, and the rest were defined as the genes retained from ancient WGD.

### Manual revision for certain genes and gene families

Anthocyanin, carotenoid, chlorophyll, isoprenoids, lipid, and sugar metabolism-related genes, as well as photosynthesis genes functionally characterized in *A. thaliana* were retrieved for their corresponding protein sequences from the Arabidopsis Information Resource (TAIR) (https://www.arabidopsis.org/index.jsp). The retrieved *A. thaliana* proteins were processed with InterProScan [94] and BLASTP [97] (i value = 1e−10) searched against mango proteins. Hits sharing > 30% amino acid identity and > 50% amino acid alignment length with the *A. thaliana* homologs were further checked for Pfam domains. Lipid genes are retrieved from the Arabidopsis Lipid Gene Database [104]. Genes involved in the photosynthesis and sugar metabolism are retrieved based on the information provided in Plant Metabolic Network [105] (https://www.plantcyc.org). Polyphenol metabolism and chlorophyll metabolism genes were retrieved based on references [41, 106]. Carotenoid biosynthesis genes were retrieved from AtIPD [107].

### Population genetics analysis

Qualified NGS reads from mango cultivars, landraces, and wild relatives (~ 20× genomic coverage for each genome) were mapped to the mango genome with Mem module in BWA (v 0.7.15, 79). The raw data of genome resequencing are deposited in the NCBI Sequence Read Archive under project ID PRJNA487154 [70]. The alignment for each genome was processed by marking duplicated reads using Picard tools (v 1.119) (https://github.com/broadinstitute/picard). The reads in insertion/deletion (Indel) regions were realigned using RealignerTargetCreator and IndelRealigner modules in the Genome Analysis Toolkit (GATK) [108]. Variant calling for each genome was carried out by GATK HaplotypeCaller to produce VCF files. All VCFs for 53 genomes were merged to single VCF file by GATK Genotype GVCFs function (v3.5). SNPs were filtered to remove variants with total a depth across genomes of < 265 (an average of 5 per genome) and depth > 2120 (approximately twice the mean depth of 20 for each genome), these with more than 2 missing genotypes. The gene-based annotation of the resulting qualified variants using ANNOVAR (v3.5c) [109].

The fourfold degenerate sites were identified according to the genomic location of gene model [110], and 370,924 SNPs located in these sites were identified and used for genetic distance calculation, the principal component analysis, and the population structure analysis. The genetic distance among each combination of 2 genomes was calculated using the equation used in heterozygous human genome [111]. A phylogenetic tree was constructed using the neighbor-joining method implemented in PHYLIP (v3.697) [112] and displayed in Evolview [113] (http://www.evolgenius.info/evolview/). The principal component analysis was conducted using EIGENSOFT 4.2 software [114]. Population structure analysis was performed with STRUCTURE [115] using the admixture and no linkage models in a burn-in length of 2000 and 2000 replicates type with 10 replicates. The best *k* (*k* = 3) was selected by Structure Harvester (v0.6.93) [116]. The STRUCTURE results with *k* values from 2 to 4 were further permuted with program CLUMPP (v1.1.2) [117] and displayed by the software Distruct (v1.1) [118].

The value of nucleotide diversity ratios (*π*) was calculated in 20-kb sliding windows and a step size of 10 kb using VariScan (v 2.0.3) [119] for the populations of *Mangifera* outgroups, populations of Southeast Asia type (*n* = 16), and India type (*n* = 8) with little or no admixture in the STRUCTURE analysis. VCFtools (v0.1.14, [120]) were used to calculate per-individual inbreeding coefficients, the regions of runs of homozygosity (ROH), and the pairwise population differentiation levels (*F*_ST_) of three populations (*Mangifera* outgroups, Southeast Asia type, and India type) with 50-kb sliding window and 20-kb step. Genome-wide high-confident SNPs were used for the calculation of nucleotide diversity ratios (*π*) and pairwise population differentiation (*F*_ST_).

## Supplementary information

**Additional file 1: Supplementary notes:** Summary of genomic survey sequencing, genome assembly, prediction and annotation of protein-coding genes.

**Additional file 2: Figure S1.** The linear relation between the genomic assembly and the genetic map of mango. **Figure S2.** Comparisons of mango proteins among five annotation evidences. **Figure S3.** Frequency distributions of synonymous substitution rates (Ks) between homologous gene pairs in syntenic blocks. **Figure S4.** The ka/ks distribution of syntenic homologous genes retained from recent WGD. **Figure S5.** Appearance of a mango fruit for the variety Alphonso, demonstrating red flush of the peel. **Figure S6.** Phylogeny of *CHS* genes in mango, sweet orange and Arabidopsis. **Figure S7.** Phylogeny of *CHS* genes in Anacardiaceae with non-Anacardiaceae *CHS*s as outgroups. **Figure S8.** Phylogeny of *CHS* genes in *Arabidopsis thaliana*, *Citrus sinensis*, *Mangifera indica*, *Pistacia vera*, *Sclerocarya birrea* and *Dimocarpus longan*. **Figure S9.** Alignment of peptide sequences of CHS in mango and Arabidopsis. **Figure S10.** The percentage of ROH (>50 kb) in mango genomes for the *Mangifera* species and different cultivars. **Figure S11.** Relationship between the genomic percentage of ROH (>50 kb) and heterozygous rate.

**Additional file 3: Table S1.** Genome survey sequencing of mango cultivates and wild relatives.

**Additional file 4: Table S2.** Summary of data used for Genome Sequencing.

**Additional file 5: Table S3.** Summary of mango genome assembly.

**Additional file 6: Table S4.** Assessment of gene coverage in mango Genome Using Trinity assembled transcripts.

**Additional file 7: Table S5.** Summary of transposable elements in mango genome.

**Additional file 8: Table S6.** The Summary of Mango Protein-coding Genes.

**Additional file 9: Table S7.** Annotation statistics of predicted proteins encoded in mango genome.

**Additional file 10: Table S8.** Plant genomes used for comparative genomics investigation.

**Additional file 11: Table S9.** Statistics of collinear genes among the blocks within mango genome.

**Additional file 12: Table S10.** Statistics of collinear blocks among chromosomes resulting from mango-specific WGD events.

**Additional file 13: Table S11.** Gene Ontology enrichment analysis of Retained Genes post-WGD.

**Additional file 14: Table S12.** Sample summary for RNA-Seq library construction.

**Additional file 15: Table S13.** Statistics of photosynthesis-related duplicated genes in mango genome.

**Additional file 16: Table S14.** Statistics of sugar metabolism-related duplicated genes in mango genome.

**Additional file 17: Table S15.** Statistics of lipid metabolism-related duplicated genes in mango genome.

**Additional file 18: Table S16.** Statistics of duplicated genes related to biosynthesis of isopentenyl diphosphate in mango genome.

**Additional file 19: Table S17.** Significant variation mango gene familes within 12 plant genomes.

**Additional file 20: Table S18**. Gene Ontology enrichment analysis of genes in significant expanded gene families.

**Additional file 21: Table S19.** Comparative analysis of disease resistance-related genes in mango and other taxonomically closely related species.

**Additional file 22: Table S20.** Publically available transcriptomic data for retrieval of CHS homologs.

**Additional file 23: Table S21.** Ks values calculated among CHS genes in mango genome.

**Additional file 24: Table S22**. Identity values of sweet orange and mango CHS peptides.

**Additional file 25: Table S23.** Frequency distribution of SNPs and Stopgain SNPs in each MAF.

**Additional file 26: Table S24**. Statistics of heterozygous rate and ROH (length >50kb) in mango genome.

**Additional file 27:** Review history.

## Data Availability

All raw sequencing reads have been deposited in the NCBI Sequence Read Archive (https://www.ncbi.nlm.nih.gov/sra) under project PRJNA487154 [70].
