# Supplementary material for: The genome evolution and domestication of tropical fruit mango
Source: Genome Biol. 2020 Mar 6;21:60. doi: 10.1186/s13059-020-01959-8 (PMC7059373; doi:10.1186/s13059-020-01959-8)
Supplement: Supplementary file 1 — Additional file 1: Supplementary notes: Summary of genomic survey sequencing, genome assembly, prediction and annotation of protein-coding genes. [file 13059_2020_1959_MOESM1_ESM.docx]

## **Supplementary Notes**

## **Genomic survey and genome size estimation**

The previous studies revealed that mango is a species with high heterozygosity both on whole genome level and transcription regions [1, 2]. Taking genome size, repeat content and genomic heterozygosity greatly affect outcome of assembly. Using NGS reads, genome survey sequencing has been carried out to select suitable mango accession for whole genome sequencing in 2014.


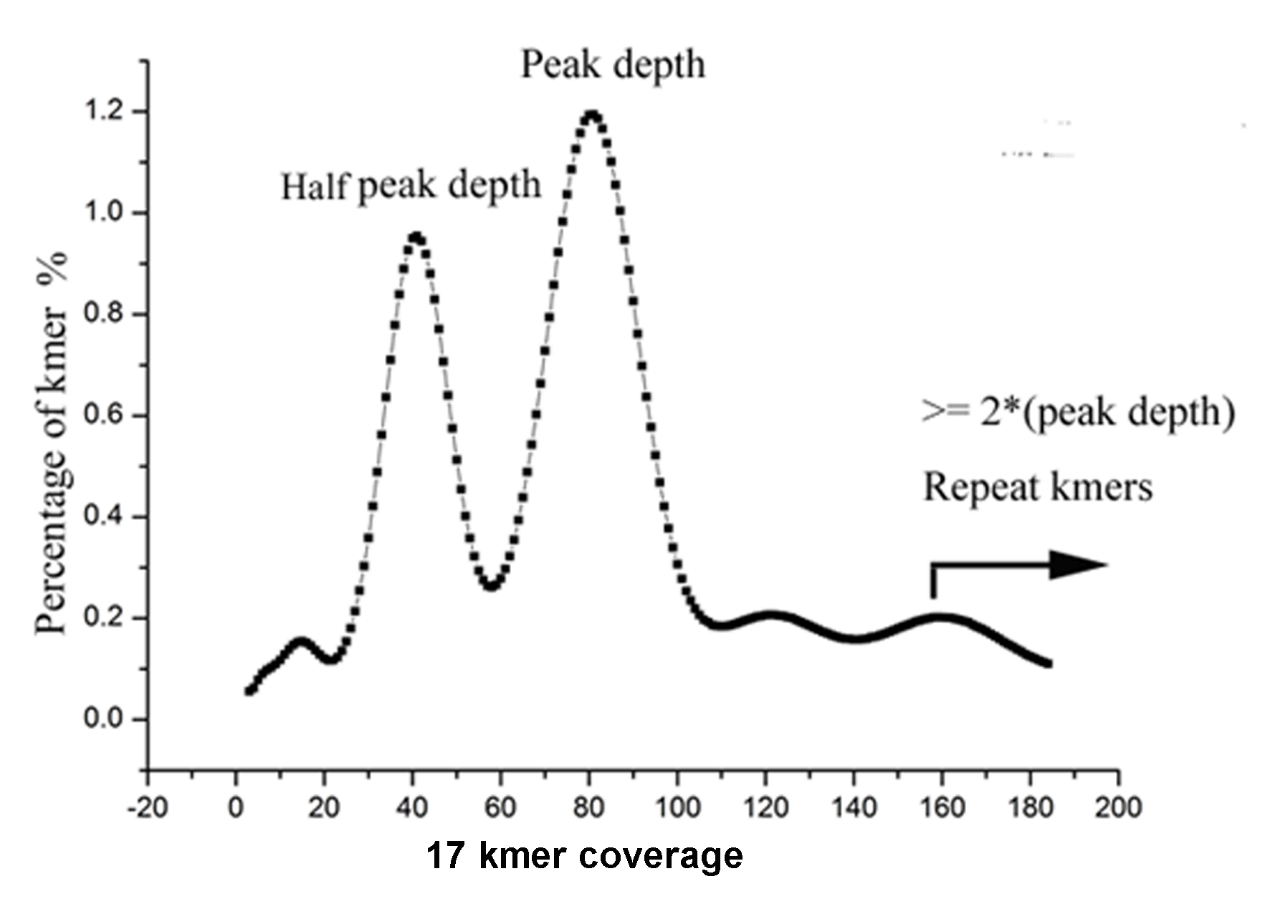


**Note Figure 1.** **The *K*-mer distribution for simulated *E. coli* genome with 2% heterozygosity rate.** Firstly, we tested the application of *k*-mer method for the estimation of genome size, repeat content and genomic heterozygosity using simulation data. Sweet orange is the species close to mango in phylogeny with genome assembly available[3]. The 310Mb size of orange assembly without continuous N sequence and larger than 10,000 bp was used as reference for simulation [3], with various combination of genomic heterozygosity (0, 1.0%, 1.5%, 1.7%, 1.85% and 2.0%) and genome sequencing coverage (10X, 20X, 40X and 80X) used. Error profile and 2×100 bp Illumina sequencing reads were simulated with Maq software (version 0.7.1) (http://maq.sourceforge.net/). Each combination of genomic heterozygosity and sequencing coverage was simulated for five times and the average values were recorded.

The genome size and repeat content were estimated using following formulas [4]: genome size = 17k-mer_Number(totally)/Peak_Depth; repeat content = 17k-mer_Number (larger than 2*Peak_Depth)/17k-mer_Number; from the simulation data with more than 20X genome coverage *k*-mer, it was clear that methods could estimate genome size and repeat content regardless of genomic heterozygosity and sequencing coverage. Coverage smaller than 20X may over-estimate genome size, especially for genomes with heterozygosity rate larger than 1.5% (**Note Figure 2**)..





**Note Figure 2. The relationship between the simulated heterozygosity rate and genome size**

**Note Table 1. The simulation and application of orange genome**

| Simulation  (heterozygoisity coverage) | Peak  depth | Kmer number | Genome size | Repeat  Kmer number | Repeat  Content (%) | Heterozygosity  index |
| --- | --- | --- | --- | --- | --- | --- |
| 2%,80x | 81 | 24656015544 | 304395254 | 8259115804 | 33.49736615 | 0.797 |
| 2%,40x | 40 | 12327984831 | 308199621 | 4295249198 | 34.84145428 | 0.802 |
| 2%,20x | 20 | 6164072160 | 308203608 | 2193311119 | 35.58217785 | 0.861 |
| 2%, 9x | 9 | 3082047521 | 342449725 | 1195453564 | 38.78764217 | 0.739 |
| 1.85%,80x | 79 | 24655673591 | 312097134 | 8050968667 | 32.65361474 | 0.668 |
| 1.85%,40x | 40 | 12327885744 | 308197144 | 4325734545 | 35.08902203 | 0.674 |
| 1.85%,20x | 20 | 6163687897 | 308184395 | 2200689481 | 35.70410309 | 0.721 |
| 1.85%,10x | 9 | 3081935494 | 342437277 | 1209088840 | 39.23147783 | 0.711 |
| 1.7%,80x | 79 | 24655615209 | 312096395 | 8651617016 | 35.08984441 | 0.611 |
| 1.7%,40x | 40 | 12328014458 | 308200361 | 4392217051 | 35.62793559 | 0.629 |
| 1.7%,20x | 20 | 6164002309 | 308200115 | 2228868945 | 36.15944371 | 0.668 |
| 1.7%, 9x | 9 | 3082042433 | 342449159 | 1223623021 | 39.70169287 | 0.681 |
| 1.5%,80x | 80 | 24655743652 | 308196796 | 8793612807 | 35.66557525 | 0.539 |
| 1.5%,40x | 40 | 12327763592 | 308194090 | 4451516510 | 36.10968426 | 0.537 |
| 1.5%,20x | 20 | 6164089164 | 308204458 | 2265237701 | 36.74894442 | 0.597 |
| 1.5%,10x | 10 | 3082014463 | 308201446 | 1154446502 | 37.45753032 | 0.716 |
| 1%,80x | 80 | 24656376352 | 308204704 | 9127204357 | 37.01762265 | 0.395 |
| 1%,40x | 40 | 12328185751 | 308204644 | 4668114514 | 37.86538107 | 0.411 |
| 1%,20x | 20 | 6163859973 | 308192999 | 2382512304 | 38.65292713 | 0.516 |
| 1%,10x | 10 | 3081920123 | 308192012 | 1202699253 | 39.02434862 | 0.581 |
| 0,80x | 80 | 24655630604 | 308195383 | 9914348827 | 40.21129691 | 0.0089 |
| 0,40x | 40 | 12327805600 | 308195140 | 5093754354 | 41.31923003 | 0.0258 |
| 0,20x | 21 | 6163816630 | 293515078 | 2500614679 | 40.56925813 | 0.172 |
| 0,10x | 11 | 3081906324 | 280173302 | 1239184561 | 40.20837854 | 0.288 |

Taking together, genome survey sequencing with coverage larger than 20X can be reliably used to predict genome size as well as repeat content and genomic heterozygosity, and make comparison between accessions using *k*-mer method with comparable genomic coverage.

|  |
| --- |



**Note Figure 3. The relationship between the simulated heterozygosity rate and heterozygosity index.** The parameter heterozygosity index was introduced to estimate the genomic heterozygosity, while heterozygosity index is 17k-mer_Number (half Peak_Depth)/17k mer_Number (Peak_Depth) [4]. We computed and compared the heterozygosity index with various heterozygosity levels on 10X, 20X, 40X and 80X coverage. Above the 20X genomic coverage, heterozygosity index is positive related to genome heterozygosity with comparable genome coverage, suggesting that heterozygosity index could reliably represent the genomic heterozygosity (**Note Figure 3**).

## **The updated of the genome size estimation**

To estimate the genome size of mango cultivar alphonso precisely with the updated methods, we used two Illumina pair-end libraries whose insert sizes are 180 bp and 500 bp, to count the kmer distribution of K=21 (Note Figure 4) and estimate the genome size and heterozygosity rate using recent released GenomeScope [5]. Meanwhile, we also run ALLPATHS-LG (version 52488) [6] to generate the genome survey report to confirm the result. The estimated genome size by GenomeScope is 356 Mb, with heterozygosity rate of 1.54%. The estimation by ALLPATHS-LG is 362 Mb with SNP rate at 1.45%. Taking the average, the estimated genome size is at about 360 Mb with the heterozygosity rate at about 1.5%.


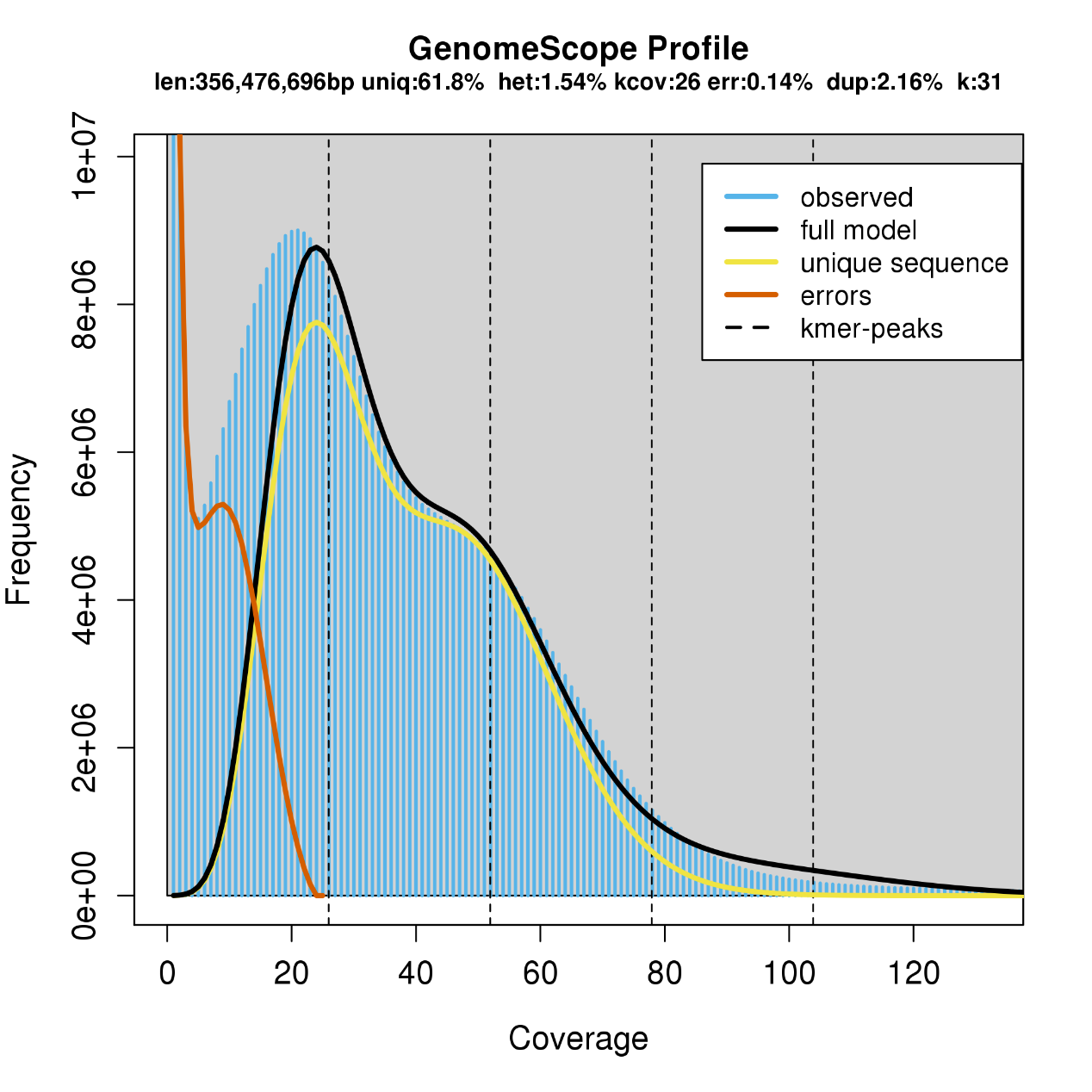


**Note Figure 4. K-mer frequency distribution and GenomeScope profile of the mango genome.** The estimated genome size is 356 Mb, with heterozygosity rate of 1.54%, which is close to estimation by ALLPATHS-LG (362 Mb, het. 1.45%).

## **Genome assembly and scaffolding with Hi-C and genetic map**

**Initial genome assembly.** To generate the initial genome assembly, a total of 5,448,383 raw reads which sum up to 86,505,121,843 bp (~216X based on 400 Mb genome size) were generated from a single cell run using PacBio Sequel II SMRT platform. The genome assembly was then generate by Canu (version 1.8) [7]. The assembly process was separated into 2 stages: Correction and Trim-Assemble. (1) Correction: running the Canu pipeline with ‘-correct’ option to cluster the reads using MHAP [8] and then generate corrected layouts using FALCON-sense [9]. These two processes were bundled within Canu (version 1.8) [7] pipline. (2) Trim-Assemble: running the Canu pipeline with ‘-trim-assemble’ option to assemble reads into contigs. To separate the haploid sequences as much as possible, we added the option ‘batOptions=-dg 3 -db 3 -dr 1 -ca 500 -cp 50’ to the assembly process according to Canu’s documentation. During this stage, we tested different parameters of overlap length from 1kb to 10kb, to find the best assembly continuity. Finally, we found that the run with 5kb overlap cutoff achieved the best N50 length (3.58 Mb at 36 contigs) of all. The assembled genome size is 624.85 Mb, which is much bigger than the estimated size. This is normal for genomes with relative high heterozygosity rate, some of the haploid contigs (haplotigs) were assembled independently thus generated some regional duplication of the genome. The initial assembly were then corrected by mapping PacBio corrected reads from stage (1) and Illumina pair-end and mate-pair short reads using minimap2 (version 2.17) [10] and corrected SNPs, INDELs and local misassembles using Pilon (version 1.23) [11]. In total, we carried out one round of the long read correction and two rounds of the short read correction on the contigs.

**Purging the haplotigs.** We used PurgeHaplotigs [12] to remove the haplotigs redundancy in the initial assembly. The PacBio raw reads were mapped to the initial assembly using minimap2 (version 2.17) [10]. After analyzing the coverage, parameters were set to ‘-l 30 -m 150 -h 270’ to remove artifacts below 30X and purge haplotigs between 30X~150X. After purging, a total of 363.08 Mb in 202 contigs were retained. The N50 size after purging is 3.86 Mb.

**Scaffolding with HiC data.** To generate near chromosome-level scaffolds, we constructed and sequenced a HiC library, which was digested by HindIII enzyme according to Berry Genomics protocol. A total of 33.77 Gb data were generated from Illumina NovaSeq platform using PE150 mode. Then we used the Jucier pipeline [13] to map the HiC data to the contigs, then carried out the assembly correction and HiC scaffolds construction using 3d-dna pipeline (version 180922) [14]. Result from 3d-dna pipeline were further visualized and manually corrected using JuiceBox [13] (biorxiv doi: 10.1101/254797) according to the “Genome Assembly Cookbook” from documentation of 3d-dna pipeline. The final HiC assembly contains 147 scaffolds and the N50 size is 18.23 Mb.

**Scaffolds integrated with genetic map.** Afterwards, the assembly was anchored with published genetic markers from 20 linkage groups [15] to construct chromosome-scale pseudo-molecules using ALLMAPS (version 1.0) [16]. A total of 6,594 markers were used to anchor scaffold to linkage groups. The markers were firstly mapped to the scaffolds using BLAT (version 34x10) [17] and the best hit was chosen for individual marker. Overall, 6,543 of 6,594 (99.22%) markers could be located onto the HiC scaffolds. In summary, the consensus map contains 20 anchored scaffolds spanning 357.44 Mb (98.44% of the HiC scaffolds) and the N50 size is 18.23 Mb.

**The refinement of final assembly.** The HiC scaffolds from previous step were further gap-filled and corrected with Pilon (version 1.22) [11] by mapping PacBio long reads with minimap2 (version 2.17) [10] and mapping Illumina pair-end reads with BWA-MEM (v0.7.15) [18]. To rescue contigs over purged in ‘**Purging the haplotigs**’ stage which may result in missing representative genes on haplotigs, we aligned unanchored genetic markers to the purged ‘haplotigs’ sequences and recovered the best hits longer than 100 kb as unplaced contigs to the assembly. The final assembly is consisted of 252 scaffolds (20 pseudo-chromosomes, 2 organelles and 230 unplaced scaffolds) that sum up to 392.98 Mb, with an N50 size of 17.65 Mb (L50 of 10 scaffolds). The GC content of the assembled scaffolds is 32.73%. The percentage of ambiguous bases (gaps) is 0.02% (82.8 kb).

**Note Figure 5. The coverage of assembled sequences by raw PacBio subreads.** The size of ‘Canu contigs’ is 624.85 Mb, which contains large percent of duplicated assembled haplotigs at 100X depth. The purging process has removed most of the duplicated haplotigs and reduced the assembly size to 363.08 Mb, which is shown as the coverage of ‘Purged contigs’. The coverage of final RC2 assembly is shown as ‘RC2 All’ for all sequences and ‘RC2 Chr’ for anchored pseudo-chromosome only. The difference is due to refinement process that rescued some unique markers contained contigs from purged to unplaced thus gained a little to the heterozygosity for ‘RC2 All’.

## **3. Prediction and Annotation of Protein-coding Genes**

Protein-coding genes were annotated using a combination of *ab initio* prediction, homologous mapping and ORF finding from transcriptome. Prior to gene annotation, repetitive sequences were masked using RepeatModeler and RepeatMasker (http://repeatmasker.org/). RepeatModeler was used to build a mango specific repeat family database by sampling and clustering repeat sequences in the genome, then the database was used by RepeatMasker to mask the repetitive sequences as lower case in the genome.

A total of 12 RNA-seq libraries from a variety of tissues, including leaf, flower, root, bark and fruit, were incorporated to construct the transcripts by mapping reads using HISAT2 (version 2.1.0) [19] and construct the transcripts using Stringtie (version 1.3.4) [20]. All constructed transcripts were combined by TACO (version 0.7.3) [21]. A sum of 123,594 *de novo* transcripts were assembled and processed as input for PASA assembly. The PASA assembler generated 115,718 isoform transcripts and identified 75,459 coding sequences (CDS) with built-in Transdecoder (PASA r20130907) [22], of which 57,355 CDS were marked as complete. The complete CDS were used as training set for BRAKER pipeline (version 2.1.4) [23]. Meanwhile, the spliced mapping results from RNA-seq data by HISAT2 (version 2.1.0) [19] were supplied to GeneMark-ES (version 2.3e) [24] to help improve the prediction. As for homologous mapping, the plant protein sequences from Uniprot database (taxonomy: 3398 [Magnoliophyta]) were manually extracted as reference and mapped to the genome using GenomeThreader [25].

Finally, all the annotation evidences were integrated using EVM (version 1.1.1) [26] and curated by ‘gffread’ tool bundled with Cufflinks (version 2.2.1) [27]. Meanwhile, repeat associated genes, including integrases, reverse transposases, were also identified using hmmscan (HMMER version 3.2.1) [28] by searching against Pfam.hmm database and removed from the evidence. Finally, a total of 41,251 protein-coding genes loci were identified, representing 112,729 isoform transcripts. The average length of the gene body and coding sequence (CDS) are 3,503 bp and 1,199 bp respectively. The average number of exons per gene, average length and average GC content of exons are 5.6 per gene, 293 bp and 41.24% respectively. As for introns, the corresponding numbers are 4.6 per gene, 424 bp and 31.98% respectively. For each locus, the longest transcript isoform was selected as the ‘primary sequence’ which was used for further analysis. Functional annotation of the genes were carried out using InterProScan (version 5.33-72.0) [29], which integrates a variety databases including TIGRFAM, Phobius, SignalP, SUPERFAMILY, PANTHER, Gene3D, ProSite, Coils, PRINTS, SMART, Pfam, PIRSF and TMHMM. The GO and KEGG accessions were also retrieved from the InterProScan result.

**References:**

1. Hirano R, Htun Oo T, Watanabe KN: **Myanmar mango landraces reveal genetic uniqueness over common cultivars from Florida, India, and Southeast Asia.** *Genome* 2010, **53:**321-330.

2. Chiang YC, Tsai CM, Chen YK, Lee SR, Chen CH, Lin YS, Tsai CC: **Development and characterization of 20 new polymorphic microsatellite markers from Mangifera indica (Anacardiaceae).** *Am J Bot* 2012, **99:**e117-119.

3. Xu Q, Chen LL, Ruan X, Chen D, Zhu A, Chen C, Bertrand D, Jiao WB, Hao BH, Lyon MP, et al: **The draft genome of sweet orange (Citrus sinensis).** *Nat Genet* 2013, **45:**59-66.

4. Liu B, Shi Y, Yuan J, Hu X, Zhang H, Li N, Li Z, Chen Y, Mu D, Fan W: **Estimation of genomic characteristics by analyzing k-mer frequency in de novo genome projects.** *arXiv preprint arXiv:13082012* 2013.

5. Vurture GW, Sedlazeck FJ, Nattestad M, Underwood CJ, Fang H, Gurtowski J, Schatz MC: **GenomeScope: fast reference-free genome profiling from short reads.** *Bioinformatics* 2017, **33:**2202-2204.

6. Gnerre S, Maccallum I, Przybylski D, Ribeiro FJ, Burton JN, Walker BJ, Sharpe T, Hall G, Shea TP, Sykes S, et al: **High-quality draft assemblies of mammalian genomes from massively parallel sequence data.** *Proc Natl Acad Sci U S A* 2011, **108:**1513-1518.

7. Koren S, Walenz BP, Berlin K, Miller JR, Bergman NH, Phillippy AM: **Canu: scalable and accurate long-read assembly via adaptive k-mer weighting and repeat separation.** *Genome Res* 2017, **27:**722-736.

8. Berlin K, Koren S, Chin CS, Drake JP, Landolin JM, Phillippy AM: **Assembling large genomes with single-molecule sequencing and locality-sensitive hashing.** *Nat Biotechnol* 2015, **33:**623-630.

9. Chin CS, Peluso P, Sedlazeck FJ, Nattestad M, Concepcion GT, Clum A, Dunn C, O'Malley R, Figueroa-Balderas R, Morales-Cruz A, et al: **Phased diploid genome assembly with single-molecule real-time sequencing.** *Nat Methods* 2016, **13:**1050-1054.

10. Li H: **Minimap2: pairwise alignment for nucleotide sequences.** *Bioinformatics* 2018, **34:**3094-3100.

11. Walker BJ, Abeel T, Shea T, Priest M, Abouelliel A, Sakthikumar S, Cuomo CA, Zeng Q, Wortman J, Young SK, Earl AM: **Pilon: an integrated tool for comprehensive microbial variant detection and genome assembly improvement.** *PLoS One* 2014, **9:**e112963.

12. Roach MJ, Schmidt SA, Borneman AR: **Purge Haplotigs: allelic contig reassignment for third-gen diploid genome assemblies.** *BMC Bioinformatics* 2018, **19:**460.

13. Durand NC, Shamim MS, Machol I, Rao SS, Huntley MH, Lander ES, Aiden EL: **Juicer Provides a One-Click System for Analyzing Loop-Resolution Hi-C Experiments.** *Cell Syst* 2016, **3:**95-98.

14. Dudchenko O, Batra SS, Omer AD, Nyquist SK, Hoeger M, Durand NC, Shamim MS, Machol I, Lander ES, Aiden AP, Aiden EL: **De novo assembly of the Aedes aegypti genome using Hi-C yields chromosome-length scaffolds.** *Science* 2017, **356:**92-95.

15. Luo C, Shu B, Yao Q, Wu H, Xu W, Wang S: **Construction of a High-Density Genetic Map Based on Large-Scale Marker Development in Mango Using Specific-Locus Amplified Fragment Sequencing (SLAF-seq).** *Front Plant Sci* 2016, **7:**1310.

16. Tang H, Zhang X, Miao C, Zhang J, Ming R, Schnable JC, Schnable PS, Lyons E, Lu J: **ALLMAPS: robust scaffold ordering based on multiple maps.** *Genome Biol* 2015, **16:**3.

17. Kent WJ: **BLAT--the BLAST-like alignment tool.** *Genome Res* 2002, **12:**656-664.

18. Li H, Durbin R: **Fast and accurate short read alignment with Burrows-Wheeler transform.** *Bioinformatics* 2009, **25:**1754-1760.

19. Kim D, Langmead B, Salzberg SL: **HISAT: a fast spliced aligner with low memory requirements.** *Nat Methods* 2015, **12:**357-360.

20. Pertea M, Kim D, Pertea GM, Leek JT, Salzberg SL: **Transcript-level expression analysis of RNA-seq experiments with HISAT, StringTie and Ballgown.** *Nat Protoc* 2016, **11:**1650-1667.

21. Niknafs YS, Pandian B, Iyer HK, Chinnaiyan AM, Iyer MK: **TACO produces robust multisample transcriptome assemblies from RNA-seq.** *Nat Methods* 2017, **14:**68-70.

22. Haas BJ, Delcher AL, Mount SM, Wortman JR, Smith RK, Jr., Hannick LI, Maiti R, Ronning CM, Rusch DB, Town CD, et al: **Improving the Arabidopsis genome annotation using maximal transcript alignment assemblies.** *Nucleic Acids Res* 2003, **31:**5654-5666.

23. Hoff KJ, Lomsadze A, Borodovsky M, Stanke M: **Whole-Genome Annotation with BRAKER.** *Methods Mol Biol* 2019, **1962:**65-95.

24. Borodovsky M, Lomsadze A: **Eukaryotic gene prediction using GeneMark.hmm-E and GeneMark-ES.** *Curr Protoc Bioinformatics* 2011, **Chapter 4:**Unit 4 6 1-10.

25. Gremme G, Steinbiss S, Kurtz S: **GenomeTools: a comprehensive software library for efficient processing of structured genome annotations.** *IEEE/ACM Trans Comput Biol Bioinform* 2013, **10:**645-656.

26. Haas BJ, Salzberg SL, Zhu W, Pertea M, Allen JE, Orvis J, White O, Buell CR, Wortman JR: **Automated eukaryotic gene structure annotation using EVidenceModeler and the Program to Assemble Spliced Alignments.** *Genome Biol* 2008, **9:**R7.

27. Ghosh S, Chan CK: **Analysis of RNA-Seq Data Using TopHat and Cufflinks.** *Methods Mol Biol* 2016, **1374:**339-361.

28. Eddy SR: **Profile hidden Markov models.** *Bioinformatics* 1998, **14:**755-763.

29. Jones P, Binns D, Chang HY, Fraser M, Li W, McAnulla C, McWilliam H, Maslen J, Mitchell A, Nuka G, et al: **InterProScan 5: genome-scale protein function classification.** *Bioinformatics* 2014, **30:**1236-1240.
