# Supplementary material for: The genome evolution and domestication of tropical fruit mango
Source: Genome Biol. 2020 Mar 6;21:60. doi: 10.1186/s13059-020-01959-8 (PMC7059373; doi:10.1186/s13059-020-01959-8)
Supplement: Supplementary file 2 — Additional file 2: Figure S1. The linear relation between the genomic assembly and the genetic map of mango. Figure S2. Comparisons of mango proteins among five annotation evidences. Figure S3. Frequency distributions of synonymous substitution rates (Ks) between homologous gene pairs in syntenic blocks. Figure S4. The ka/ks distribution of syntenic homologous genes retained from recent WGD. Figure S5. Appearance of a mango fruit for the variety Alphonso, demonstrating red flush of the peel. Figure S6. Phylogeny of CHS genes in mango, sweet orange and Arabidopsis. Figure S7. Phylogeny of CHS genes in Anacardiaceae with non-Anacardiaceae CHSs as outgroups. Figure S8. Phylogeny of CHS genes in Arabidopsis thaliana, Citrus sinensis, Mangifera indica, Pistacia vera, Sclerocarya birrea and Dimocarpus longan. Figure S9. Alignment of peptide sequences of CHS in mango and Arabidopsis. Figure S10. The percentage of ROH (>50 kb) in mango genomes for the Mangifera species and different cultivars. Figure S11. Relationship between the genomic percentage of ROH (>50 kb) and heterozygous rate. [file 13059_2020_1959_MOESM2_ESM.docx]

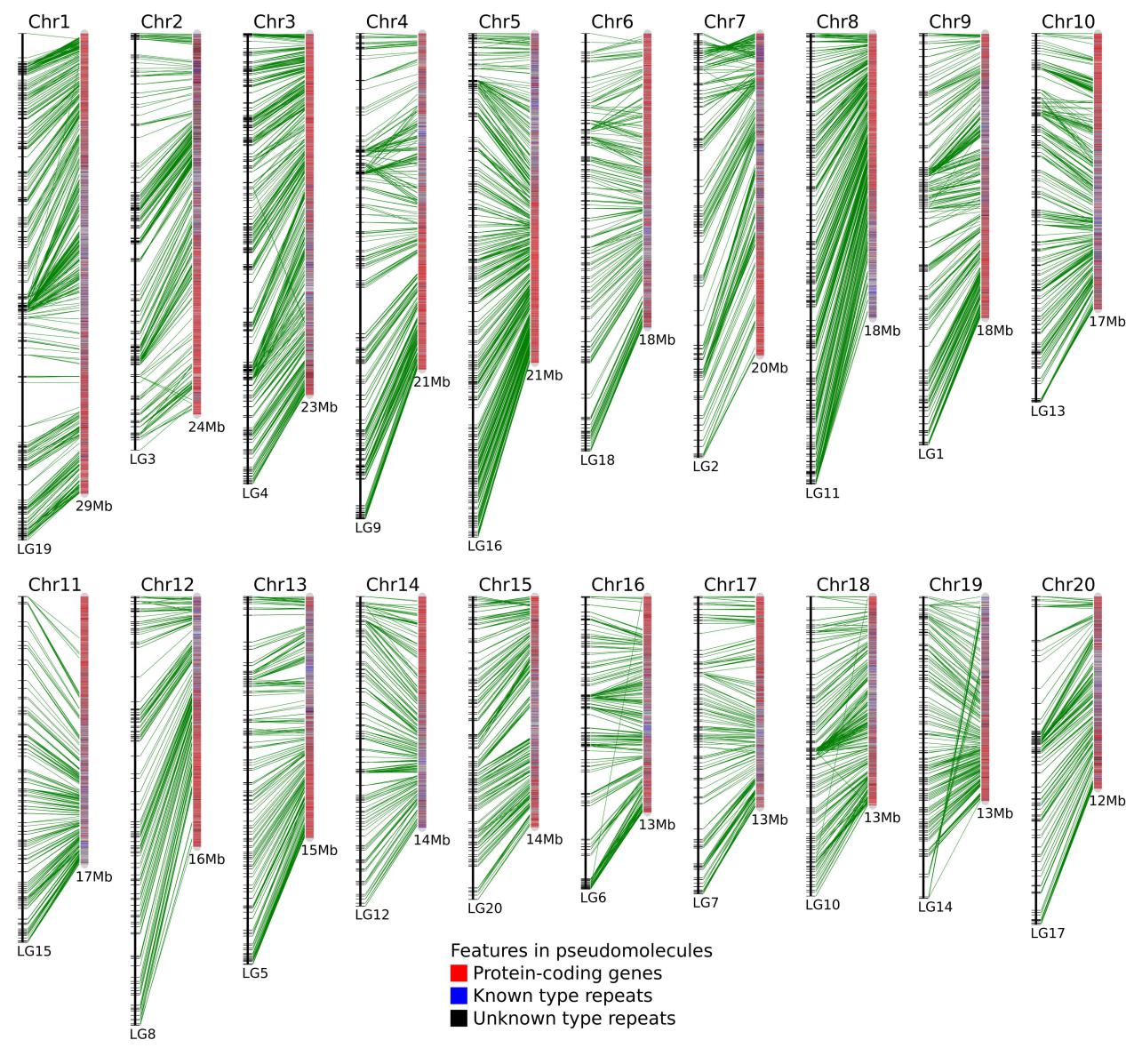


**Figure S1. The linear relation between the genomic assembly and the genetic map of mango.**

Mango scaffolds were anchored in the twenty linkage groups with the unique mapped genetic markers (blue bars). The pseudo-chromosome number are assigned on the basis of the estimated length of the genetic linkage groups.


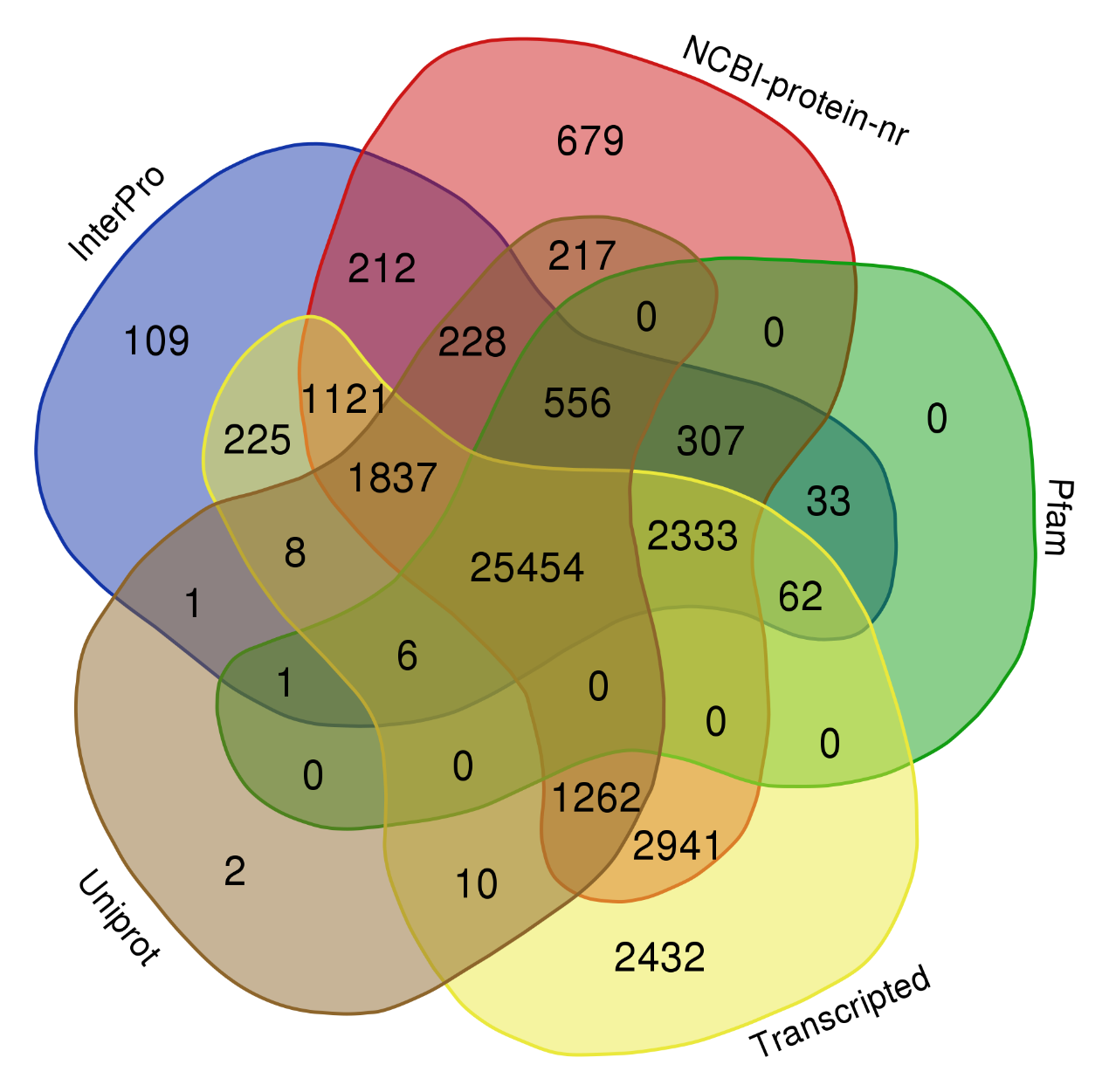


**Figure S2. Comparisons of mango proteins among five annotation evidences.**





**Figure S3. Frequency distributions of synonymous substitution rates (Ks) between homologous gene pairs in syntenic blocks of mango-mango, longan-longan, orange-orange, mango-orange, mango-longan and orange-longan, respectively.**





**Figure S4. The ka/ks distribution of syntenic homologous genes retained from recent WGD.**


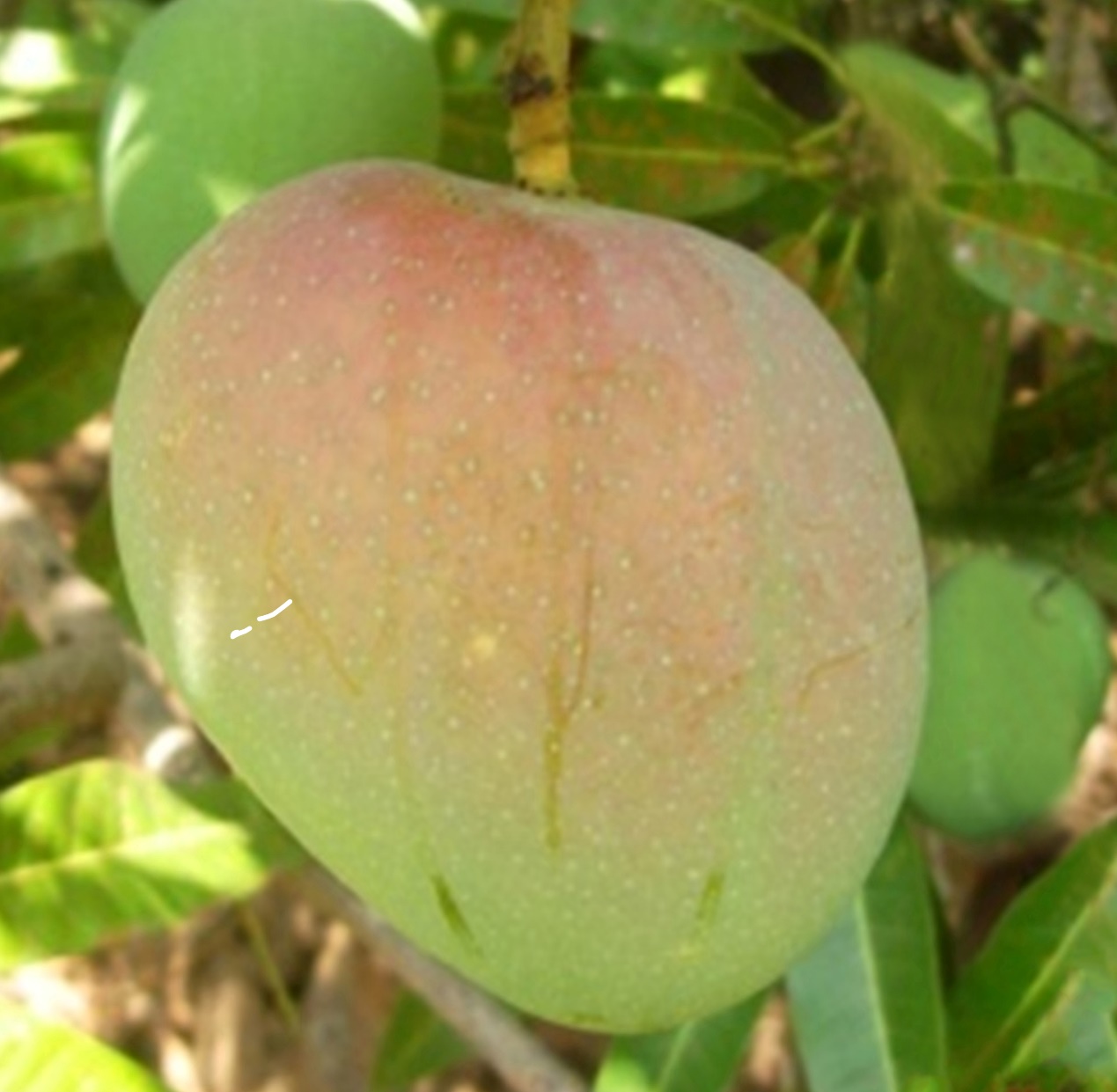
**Figure S5. Appearance of a mango fruit for the variety Alphonso, demonstrating red flush of the peel.**


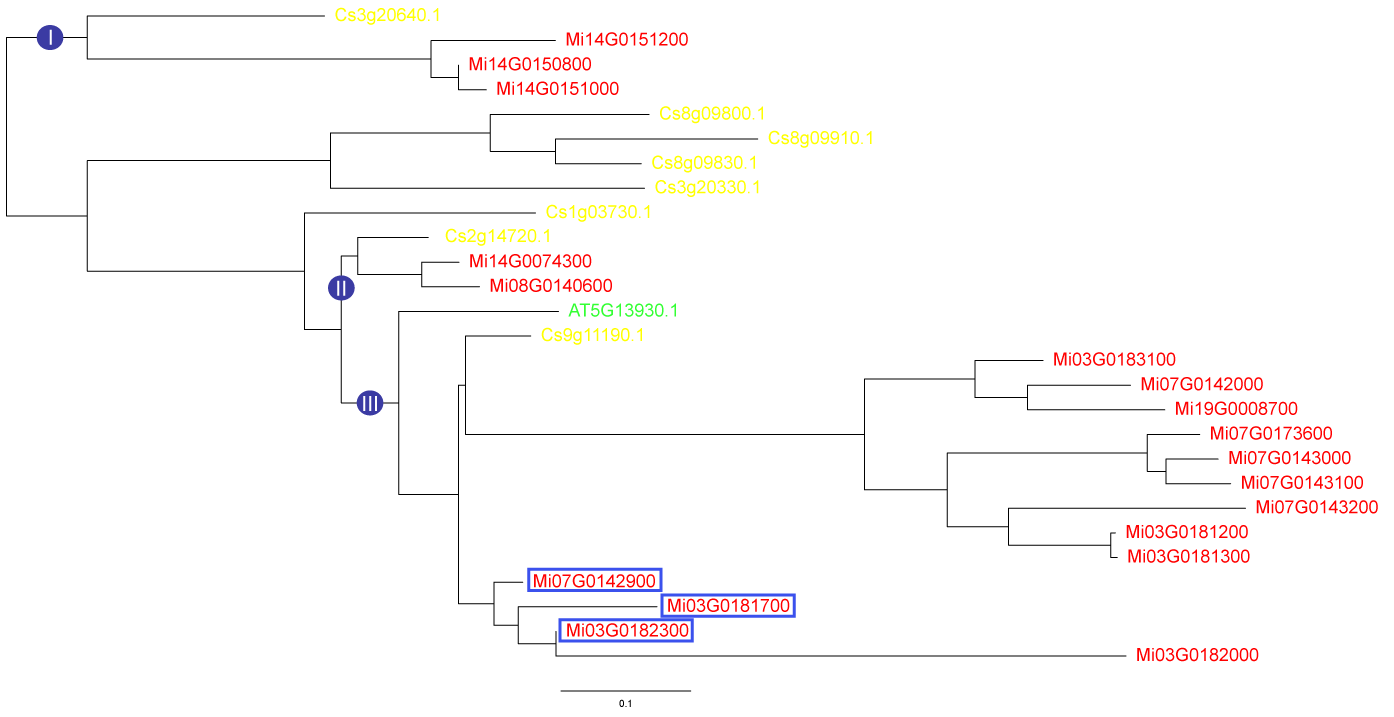


**Figure S6. Phylogeny of *CHS* genes in mango, sweet orange and Arabidopsis.**

Peptide sequences were aligned with MUSCLE and phylogeny was reconstructed by MrBAYES and PHYML, respectively. The three major clades are demonstrated, namely I, II and III, respectively. Boxed IDs represent putative pseudogenes.

A

**
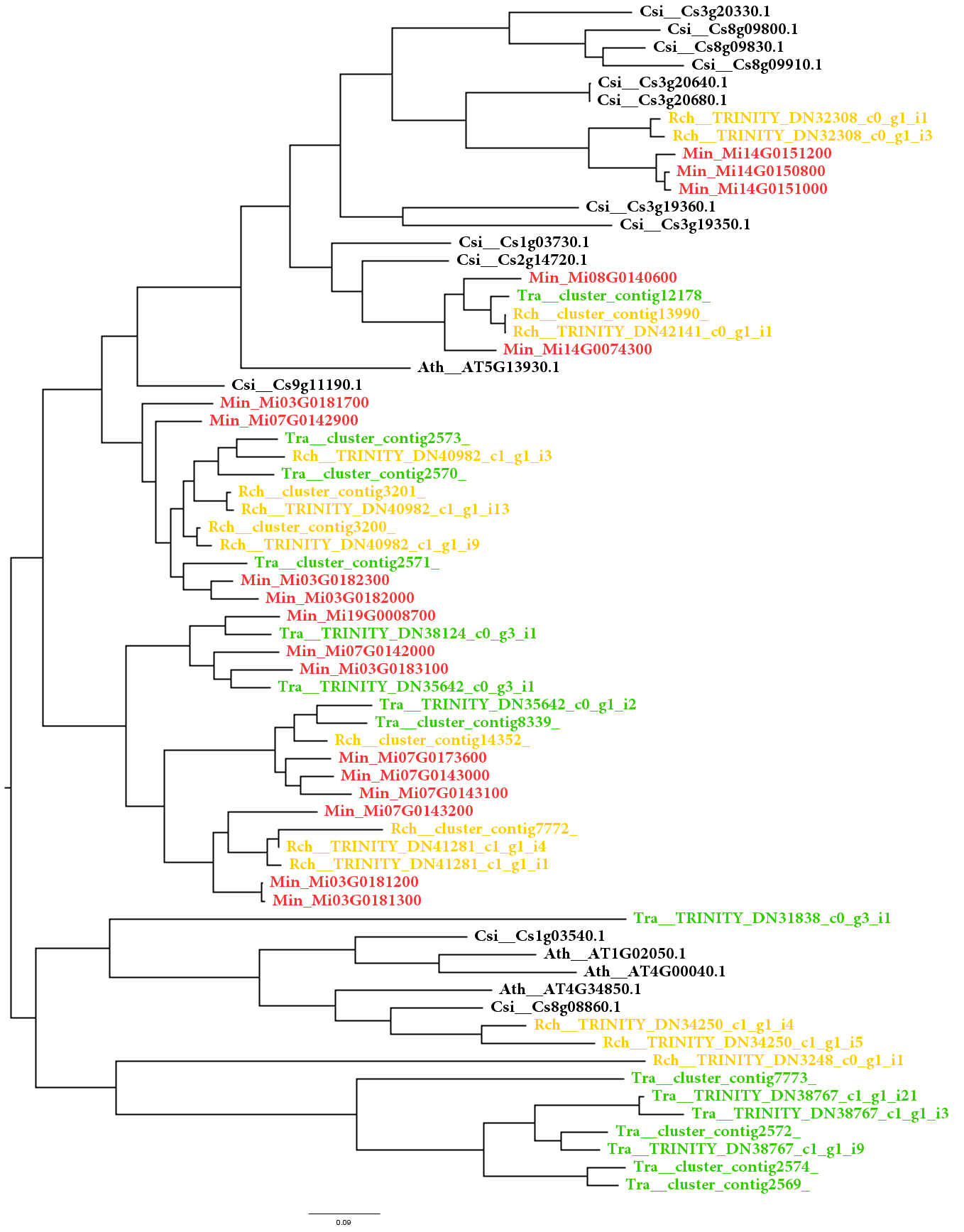
**

B

**
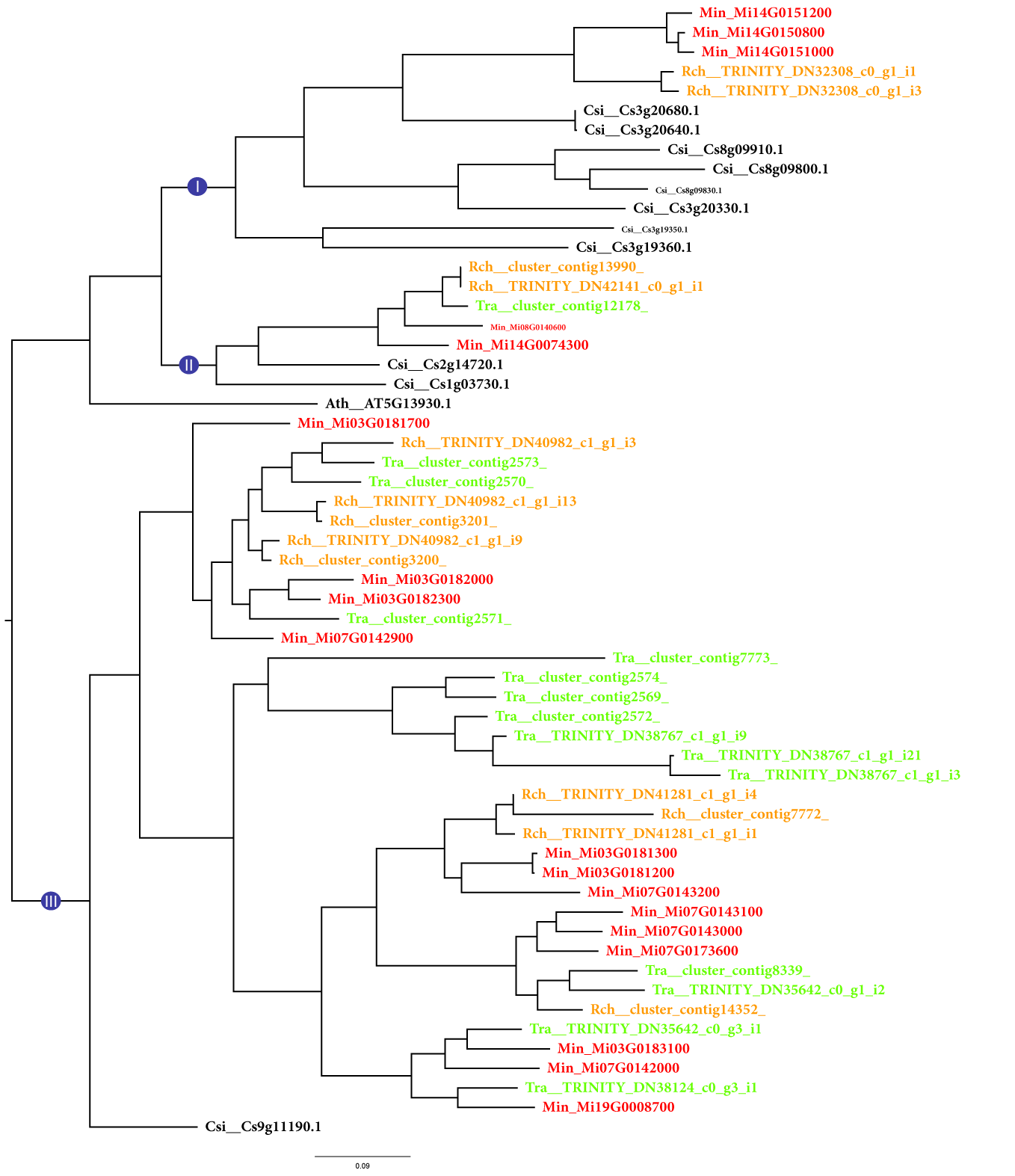
**

**Figure S7. Phylogeny of *CHS* genes in Anacardiaceae with orange and Arabidopsis *CHS*s as outgroups.**

CDSs of mango CHSs, and contig sequences of *CHS*s from transcriptomic assembly of *Rhus chinensis* and *Toxicodendron radicans* (both belonging to Anacardiaceae as mango), together with CDSs of Arabidopsis and sweet orange CHSs were aligned with MUSCLE, and phylogeny were reconstructed by MrBAYES and PHYML, respectively. (A) Phylogeny of all the CHS family sequences. (B) Phylogeny of *CHS* sequences with *CHSL* genes removed. Three major clades are labeled as I, II, and III, respectively as in Fig. 3C.


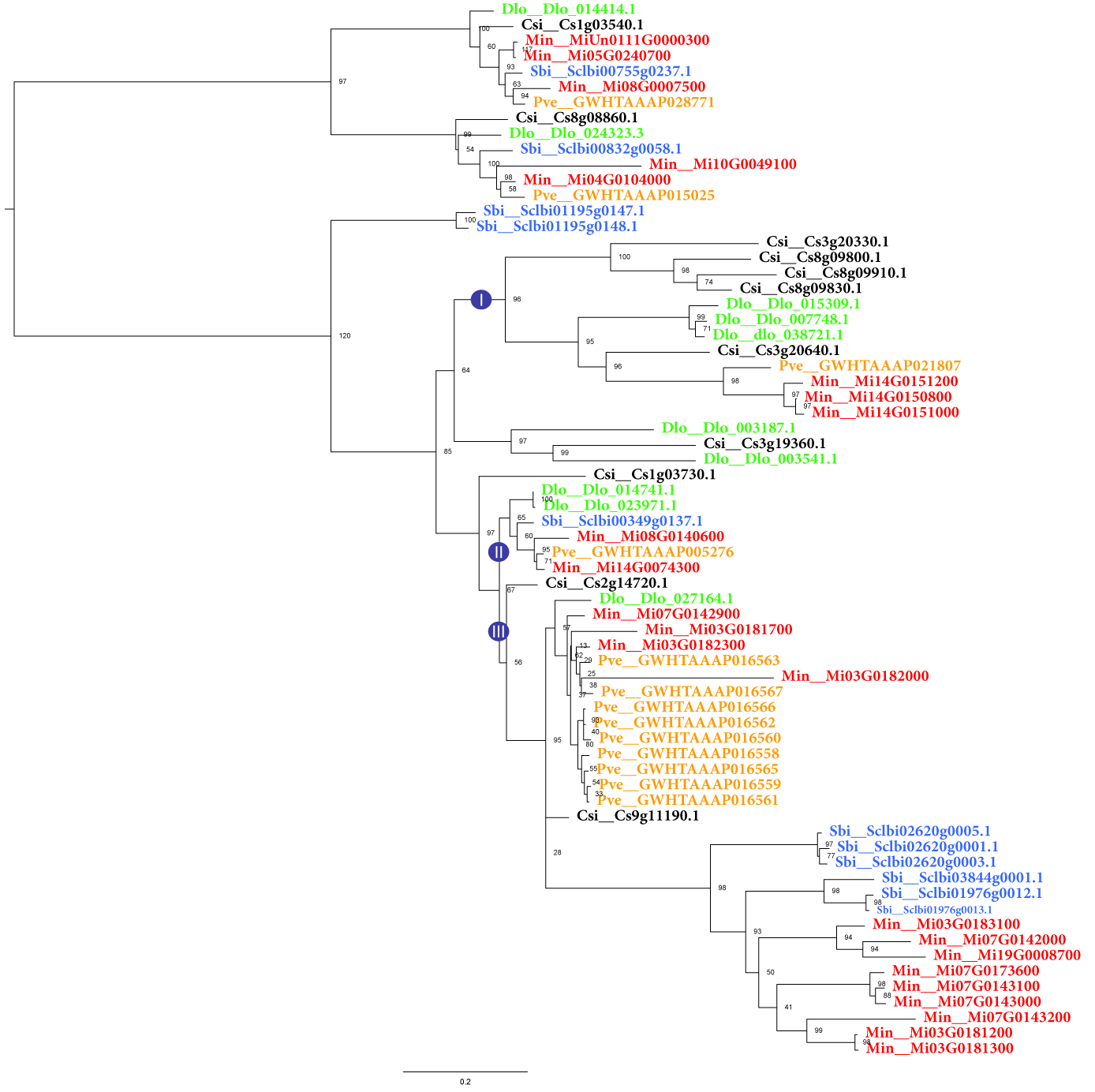


**Figure S8. Phylogeny of *CHS* genes in *Arabidopsis thaliana*, *Citrus sinensis*, *Mangifera indica*, *Pistacia vera*, *Sclerocarya birrea* and**

***Dimocarpus longan*. Three major clades are labeled as I, II, and III, respectively as in Fig. 3C and Figure 7B.**


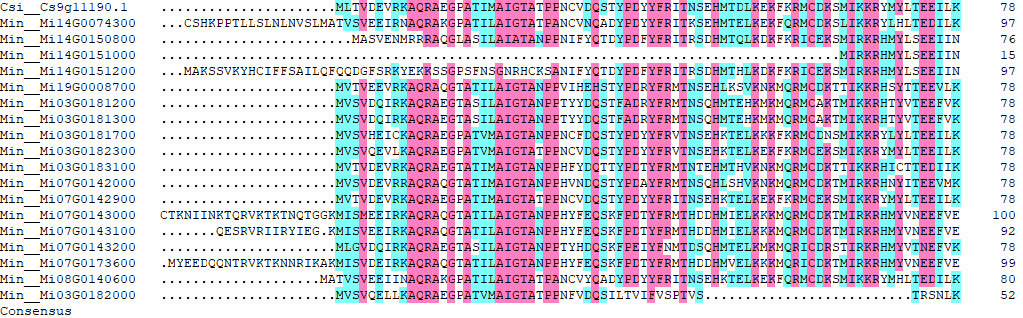


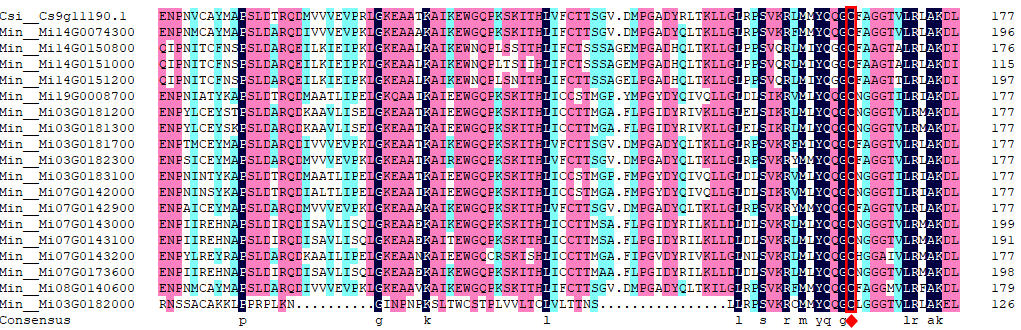


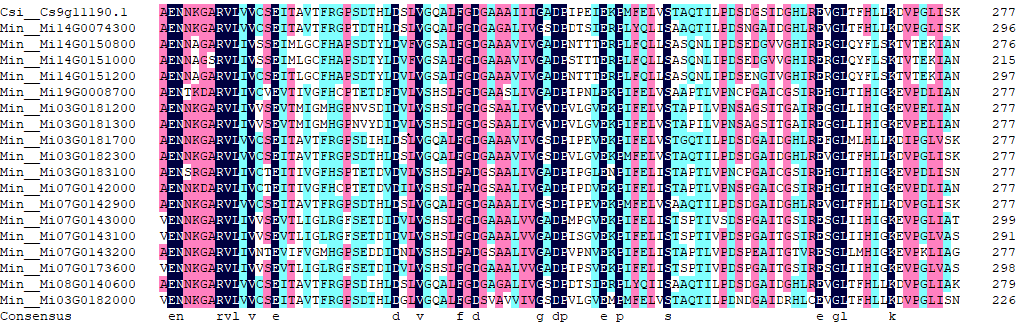


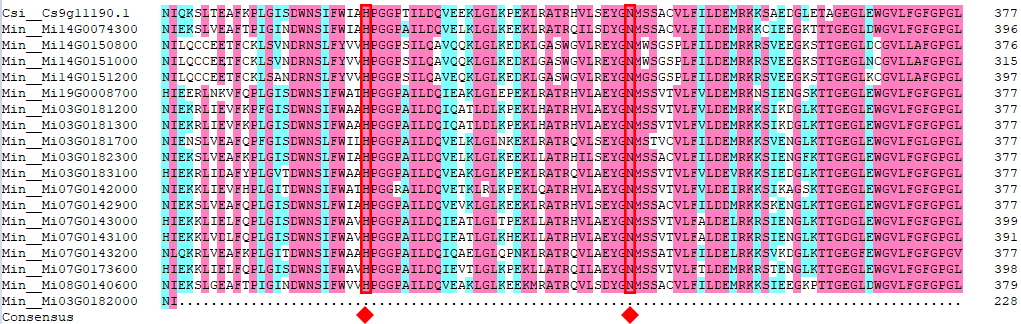


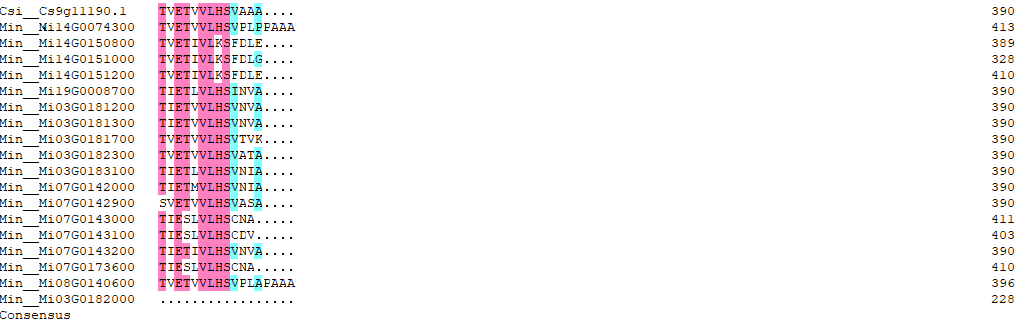


**Figure S9. Alignment of peptide sequences of CHS in mango and Arabidopsis.**

Boxed sites represent conserved residues which are essential for CHS activities.





**Figure S10. Percentage of ROH (>50 kb) in mango genomes for the *Mangifera*** **species and different cultivars.**


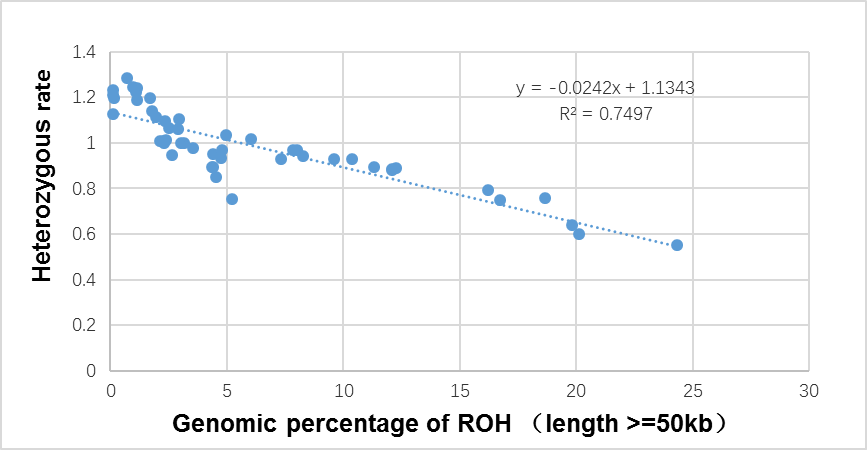


**Figure S11. Relationship between the genomic percentage of ROH (>50 kb) and heterozygous rate.**
