# Supplementary material for: The genome evolution and domestication of tropical fruit mango
Source: Genome Biol. 2020 Mar 6;21:60. doi: 10.1186/s13059-020-01959-8 (PMC7059373; doi:10.1186/s13059-020-01959-8)
Supplement: Supplementary file 27 — Additional file 27: Review history. [file 13059_2020_1959_MOESM27_ESM.docx]

Review History

**First round of review**

**Reviewer 1**

**Are you able to assess all statistics in the manuscript, including the appropriateness of statistical tests used?**

Yes, and I have assessed the statistics in my report.

**Comments to author:**

In this MS, the authors generated a high-quality genome assembly for one important tropical fruit, mango. Comparative genomic analysis reveals a recent WGD event and expansion of CHS genes in mango. The genes involved in photosynthesis and lipid metabolism are preferentially retained after the WGD event. Moreover, genome resequencing of 21 diverse accessions reveals unique genetic background of South China landraces. These data and information provide an invaluable resource for mango genetic research and molecular breeding. I have some specific comments as below.

Major comments:

1. In the family Anacardiaceae, the genome sequences of two species (pistachio and cashew) have been released. For the genome evolution and comparative analysis sections, the genomes of pistachio and cashew should be included. Is the recent WGD specific in mango?

2. The authors inferred two possible routes of tandem duplications of CHS genes in Chr. 7 and Chr.3. I suggest they provide more evidences or explain well about this, especially for the occurrence time of duplicated genes.

3. The detailed statistics of the evaluation of the genome assembly based on CEGMA and BUSCO should be included in the table or supplementary data.

4. The author described the specific roles of Clade I CHSs among four cultivars, but the phenotype of Alphonso was missed in Fig 4a. In addition, the authors showed the genes in Clade II have similar gene expression pattern in different varieties of fruit peels and vegetative tissues (Fig. 3D), but actually the expression patterns are different not only in different tissues but also among different cultivars.

5. In the section of discussion and conclusion, "the first reference genome for the Anacardiaceae family " should be removed.

6. The authors detected the expansion event of CHS gens in Anacardiaceae, but with only two species and transcriptome data. More data are needed.

7. In germplasm diversity part, "Indica type and Southeast Asia type varieties fall into two independent groups" can not be an evidence that "mango cultivars have two centers of genetic diversity".

8. For the Mango germplasm diversity studies, authors declared the commercial varieties bred in China show strong allelic admixture from India and Southeast Asia type germplasm. However, not all commercial varieties have strong allelic admixture. Moreover, the strong admixture pattern was not detected when K=3.

9. Authors should give more explanation why Chinese landraces are far from other varieties. It is better to know which genes or genomic regions can be useful genetic resources in the mongo breeding in future.

Minor comments:

1. Genome assembly and annotation part, please make sure the assembly genome size is 398.6 Mb or 396.8 Mb.

2. Please give the size information for each chromosomes (pseudomolecules).

**Reviewer 2**

**Are you able to assess all statistics in the manuscript, including the appropriateness of statistical tests used?**

No, I do not feel adequately qualified to assess the statistics.

**Comments to author:**

The manuscript entitled " The genome evolution and domestication of tropical fruit mango " looks to present the first genome assembly for tropical fruit mango. The authors then analyzed the whole-genome duplications, the expansion of gene families and germplasm genomic diversity, etc. The quality of the mango tree genome was much improved compared with the previous assemblies. However, there is a serious problem in the genome assembly that the authors overlooked, which made many of the analytical results be suspicious. All results should be verified after the assembly is further improved.

Major problems:

1．	The contiguity and quality of the genome assembly are quite poor and problematic. The genome assembly was basically sequenced and assembled based on a combination of Illumina pair-end short reads and mate-pair short reads. Authors performed a hybrid assembly strategy by adding SMRT long reads (~8.93 Gb, ~20X), but a number of studies have indicated that this is not very helpful to guarantee the assembly accuracy, as shown by a short Contig N50 = 41.7kb. As a relatively small genome size (418 Mb), the assembly result is quite fragmented. Although authors anchored them into 20 linage groups with the help of genetic maps, to be honest, I think that the quality of genome assembly is problematic using NGS platforms to sequence such a small genome nowadays. Authors state that this is a reference genome, but I do not think that it has reached the level.

2．	Given a relatively small genome size (418 Mb) and highly heterozygous mango genome (see Supplementary Fig. 1), it is really necessary to perform long SMRT sequencing Sequel II platform (subreads N50: 25K) to generate a high-quality reference genome assembly. Given such a small but highly heterozygous genome, it is recommended to generate 200X data (~80 Gb), which just needs to spend under $3K. The expected diploid assembly size is likely to be >400 Mb if enough data were used. The assembly that the authors present here is actually a mixture of two diploid genomes, which include both heterozygous and duplicated sequences. To address the above issue, the authors should use Falcon-unzip (or a software with similar functions) to obtain a (full) haploid genome (a mixture of the two parental haploids is OK but should be with minimal sequence redundancy). The analyses of WGD, gene family expansion, genome resequencing, and so on, should be redone based on the haploid genome (instead of the current mixture of diploids). It is always basic requirement to generate a really high-quality genome assembly to ensure subsequent data analyses and those novel findings are authentic.

3．	Only based on resequencing 21 genomes of mango varieties, authors try to demonstrate the existence of a landrace group distinct to backgrounds of mango varieties from two centers of genetic diversity that have long been proposed. I cannot reject these authors' findings, but results they reported here can not persuade me at all due to too small sample size. Given globally abundant mango germlasm and cultivars, I strongly suggest to reasonably increase the sample size to reach a convincing conclusion.

**Authors Response**

**Point-by-point responses to the reviewers’ comments:**

Reviewer comments and replies:

Reviewer #1: In this MS, the authors generated a high-quality genome assembly for one important tropical fruit, mango. Comparative genomic analysis reveals a recent WGD event and expansion of CHS genes in mango. The genes involved in photosynthesis and lipid metabolism are preferentially retained after the WGD event. Moreover, genome resequencing of 21 diverse accessions reveals unique genetic background of South China landraces. These data and information provide an invaluable resource for mango genetic research and molecular breeding. I have some specific comments as below.

Major comments:

1. In the family Anacardiaceae, the genome sequences of two species (pistachio and cashew) have been released. For the genome evolution and comparative analysis sections, the genomes of pistachio and cashew should be included. Is the recent WGD specific in mango?

*Reply: We noticed that an early-released version of cashew genome was available online (https://phytozome.jgi.doe.gov/) when we prepared our manuscript. We really detected major Ks peaks for cashew vs. cashew and cashew and mango, which likely occurred after split of Anacardiaceae and Sapindaceae (longan), suggesting WGD was at least shared by mango and cashew (see figure below). As the genome data were not yet formally published in a scientific literature, we did not include the result in the manuscript. In the revision stage, to respond to the reviewer’s concern, we contacted Drs. Dario Grattapaglia and Orzenil Silva-Junior, the principal investigators of the cashew genome project. Unfortunately, we failed to obtain permit to include results in our manuscript based on analysis of the cashew genome.*

*After we submitted our manuscript, we noticed that pistachio genome was released. In the revision stage, we included the pistachio genome into our analyses. We failed to detect a Ks peak in pistachio genome after the split of Anacardiaceae and Sapindaceae, in agreement with the results shown in the article by Zeng et al. (Zeng et al., Genome Biology 20:79,2019) (see the figure below). The result indicates that the WGD was specific to mango/cashew and was not shared by pistachio. However, we cannot rule out possibility that the fragmented pistachio genome assembly (contig N50 size of 75.7kb and scaffolded N50 of 949.2kb) led to incomplete prediction of gene models.*

2. The authors inferred two possible routes of tandem duplications of CHS genes in Chr. 7 and Chr.3. I suggest they provide more evidences or explain well about this, especially for the occurrence time of duplicated genes.

*Reply: We feel the possible routes of tandem duplications we suggested are not precise, and thus we removed Fig. 3F from the manuscript. Also we added synteny analysis of the whole chromosomes of chr. 3, chr. 7 of mango and sweet orange chr. 9, and updated microsynteny analysis of the regions harboring CHSs as likely results of tandem duplication.*

3. The detailed statistics of the evaluation of the genome assembly based on CEGMA and BUSCO should be included in the table or supplementary data.

*Reply: The CEGMA and BUSCO percentage data are included in the updated Table 1. Also updated are data for contigs and statistics of the new genome assembly.*

4. The author described the specific roles of Clade I CHSs among four cultivars, but the phenotype of Alphonso was missed in Fig 4a. In addition, the authors showed the genes in Clade II have similar gene expression pattern in different varieties of fruit peels and vegetative tissues (Fig. 3D), but actually the expression patterns are different not only in different tissues but also among different cultivars.

*Reply: We have put a picture of the variety Alphonso in the additional file (Figure S5). We think it is better to put it in the supplementary file rather than in Fig. 4a, as no expression data are in Fig. 4. We agree with the reviewer about the difference among different cultivars, and we have changed our interpretation in the Results section.*

5. In the section of discussion and conclusion, "the first reference genome for the Anacardiaceae family " should be removed.

*Reply: We have removed these sentences.*

6. The authors detected the expansion event of CHS gens in Anacardiaceae, but with only two species and transcriptome data. More data are needed.

*Reply: We have added the analysis of CHS from genomes of Pistacia vera and Sclerocarya birrea, both belonging to Anacardiaceae.*

7. In germplasm diversity part, "India type and Southeast Asia type varieties fall into two independent groups" can not be an evidence that "mango cultivars have two centers of genetic diversity".

*Reply: Extensive sampling of varieties, landrace and wild plants from diverse geographic regions is a prerequisite to clear clarification of mango genetic diversity. We only have access to limited germplasms, but our results clarified two groups of mango varieties, discovered allelic admixture in commercial varieties and showed distinct genetic background of landraces. We revised our interpretation (This coincides with the long proposed two centers of domestication, one in India and the other in continental Southeast Asia).*

8. For the Mango germplasm diversity studies, authors declared the commercial varieties bred in China show strong allelic admixture from India and Southeast Asia type germplasm. However, not all commercial varieties have strong allelic admixture. Moreover, the strong admixture pattern was not detected when K=3.

*Reply: We sequenced additional commercial cultivars and Chinese landraces. Using the updated genetic diversity data based on improved genome version, the Chinese landrace could be classified as a distinct group, while the commercial varieties bred in China represented variated and complex allelic admixture from India and Southeast Asia type germplasm especially when K=4. We agree with the reviewer’s concern. Based on our updated data, some varieties such as Alphonso and Mulgoba, which are also concerned traditional germplasms, do not show any allelic admixture. We updated the interpretation in the Results section.*

9. Authors should give more explanation why Chinese landraces are far from other varieties. It is better to know which genes or genomic regions can be useful genetic resources in the mongo breeding in future.

*Reply: To address the reviewer’s concern, we resequenced 27 additional germplasms including 9 more landrace germplasms collected from South China, and mapped the reads to the updated genome assembly. Based on the updated data, the Chinese landraces don’t form an independent group. They rather have more close relationships with Southeast Asia germplasms, although demonstrating distinct clades. Also, we heavily revised our interpretation in the Abstracts, Results and Discussion sections.*

*We tried to identify genes with the genetic diversity declined in two mango centers using the wild relatives as groups and the group specific regions between two centers. However, we failed to identify significantly enriched functional roles in genes although we tried with different parameters.*

Minor comments:

1. Genome assembly and annotation part, please make sure the assembly genome size is 398.6 Mb or 396.8 Mb.

*Reply: The assembly size of updated version is 392.6 Mb. We have checked it for the consistency in the revised manuscript.*

2. Please give the size information for each chromosomes (pseudomolecules).

*Reply: The size information for each pseudomolecule is included in Figure S1 and Table S10 of the supplemental materials.*

Reviewer #2: The manuscript entitled " The genome evolution and domestication of tropical fruit mango " looks to present the first genome assembly for tropical fruit mango. The authors then analyzed the whole-genome duplications, the expansion of gene families and germplasm genomic diversity, etc. The quality of the mango tree genome was much improved compared with the previous assemblies. However, there is a serious problem in the genome assembly that the authors overlooked, which made many of the analytical results be suspicious. All results should be verified after the assembly is further improved.

Major problems:

1． The contiguity and quality of the genome assembly are quite poor and problematic. The genome assembly was basically sequenced and assembled based on a combination of Illumina pair-end short reads and mate-pair short reads. Authors performed a hybrid assembly strategy by adding SMRT long reads (~8.93 Gb, ~20X), but a number of studies have indicated that this is not very helpful to guarantee the assembly accuracy, as shown by a short Contig N50 = 41.7kb. As a relatively small genome size (418 Mb), the assembly result is quite fragmented. Although authors anchored them into 20 linage groups with the help of genetic maps, to be honest, I think that the quality of genome assembly is problematic using NGS platforms to sequence such a small genome nowadays. Authors state that this is a reference genome, but I do not think that it has reached the level.

*Reply: We appreciate the reviewer’s advice. To address the reviewer’s concerns, we generated 86.5Gb (~240X) single-molecule subreads using PacBio Sequel II platform, and de novo assembled the genome using CANU with parameters set for genomes with high heterozygous rates. After correcting and polishing with paired-end and mate-paired short reads, we obtained an improved version of the assembly. As compared to 19426 contigs we obtained with N50 size of 41.7kb for the previous version, the updated assembly only has 420 contigs with N50 size of 3.5Mb (See table below). In addition, we generated HiC data to facilitate generating chromosome-level super-scaffolds and correcting mis-assemblies. Based on the updated assembly, we re-annotated the genome and performed all the analyses again.*

*We generated NGS data using Illumina platform and produced low-coverage third-generation sequencing data in 2014. Back at that time, the PacBio platform was at the beginning stage, and it was very costly to perform sequencing on it. We agree with the reviewer on the contiguity and quality of the genome we submitted previously. As indicated by the features shown in the table below, the quality of the current genome assembly is greatly improved, and at least comparable to those of the high quality genome assembly recently published with similar genome size and complexity (e.g. Chen et al, Nat Gen 51:1549, 2019; Zeng et al, Genome Biol 20:79, 2019; Li et al, Nat Plants 5:174, 2019).*

*Class Name Version v1.0 Version Mi_RC2*

*Genome Assembly*

*Assembly strategy NGS+PacBio (10x)*

*+genetic map PacBio(240X) +HiC+NGS*

*+genetic map*

*Scaffold N50 size*

*(Initial assembly) 1.34 Mb 3.5 Mb*

*Scaffold number 595 252*

*Contig number 19,426 420*

*Contig N50 size 41.7 kb 3.5 Mb*

*Gaps size (%) 29.9 Mb (7.5%) 82.8 kb (0.02%)*

*Genome evaluation Genetic marker 6,523 (98.2%) 6,543 (99.2%)*

*Complete BOSCOs 95.1% 95.9%*

*Missing BOSCOs 3.2% 2.6%*

*Protein-coding genes Proteins number 38, 058 41,251*

*Proteins number with annotation evidence 36,168 39,473*

*CAD 9 11*

*PGT1 8 15*

*CAO 11 18*

*FECH 23 37*

*CAS1 15 18*

*(CC)-NBS-LRR 214 328*

*(TIR)-NBS-LRR 48 109*

*RLK 90 104*

*RLP 113 192*

*CHS 22 23*

2． Given a relatively small genome size (418 Mb) and highly heterozygous mango genome (see Supplementary Fig. 1), it is really necessary to perform long SMRT sequencing Sequel II platform (subreads N50: 25K) to generate a high-quality reference genome assembly. Given such a small but highly heterozygous genome, it is recommended to generate 200X data (~80 Gb), which just needs to spend under $3K. The expected diploid assembly size is likely to be >400 Mb if enough data were used. The assembly that the authors present here is actually a mixture of two diploid genomes, which include both heterozygous and duplicated sequences. To address the above issue, the authors should use Falcon-unzip (or a software with similar functions) to obtain a (full) haploid genome (a mixture of the two parental haploids is OK but should be with minimal sequence redundancy). The analyses of WGD, gene family expansion, genome resequencing, and so on, should be redone based on the haploid genome (instead of the current mixture of diploids). It is always basic requirement to generate a really high-quality genome assembly to ensure subsequent data analyses and those novel findings are authentic.

*Reply: As interpreted above, we produced ~240x long SMRT sequences, and we performed HiC sequencing. Initially, we obtained an assembly of 624.85Mb, about 1.73X of the estimated genome size; then we purged the haplotigs using PurgeHaplotigs, which reduced the assembly to 363.08Mb. This was very close to the estimated haploid genome size. The details of genome assembly are interpreted in the updated supplementary Notes (Additional file 1).*

*As showed in the following coverage plot, about 75% of the assembly before purging was half-covered, which is probably from separately assembled haplotigs. However, about 25% of the assembly is still ‘stick together’ that could not be fully separately assembled, which prevents us from generating a full haploid assembly. Further, the purging process could purge most (~90%) of the duplicated haplotigs, but not 100% of them. When we checked the purged haplotigs, we found that some genetic markers and conserved BOSCO genes were unique in either side of the haploid genomes. To avoid missing critical information, we chose to rescue some haplotigs from purged content back to the unplaced content which might introduce sequence redundancy. The assembled pseudo-chromosome was considerably resembled for a haploid genome, which was further used for reanalyzing. To rule out possible redundancy from unplaced content, the analysis of WGD and gene family expansion were based on genes located in 20 chromosomes. The updated results, such as detection of a recent WGD event, the prevalent retention of homologous genes after WGD, the CHS expansion as results of WGD and tandem duplication, and the function enrichment of expanded gene families were comparable with that based on the version v1.0. The unplaced content was mainly designated for a reference of dispersible genes.*

*The coverage of assembled sequences by raw PacBio subreads. The size of ‘Canu contigs’ is 624.85 Mb, which contains large percentage of duplicated assembled haplotigs at 100X depth. The purging process removed most of the duplicated haplotigs and reduced the assembly size to 363.08 Mb, which is shown as the coverage of ‘Purged contigs’. The coverage of final RC2 assembly is shown as ‘RC2 All’ for all sequences and ‘RC2 Chr’ for anchored pseudo-chromosome only. The difference is due to refinement process that rescued some unique markers containing contigs from purged to unplaced thus gained a little to the heterozygosity for ‘RC2 All’.*

3． Only based on resequencing 21 genomes of mango varieties, authors try to demonstrate the existence of a landrace group distinct to backgrounds of mango varieties from two centers of genetic diversity that have long been proposed. I cannot reject these authors' findings, but results they reported here can not persuade me at all due to too small sample size. Given globally abundant mango germlasm and cultivars, I strongly suggest to reasonably increase the sample size to reach a convincing conclusion.

*Reply: We thank the reviewer for pointing this out, and we agree with the reviewer on the sample size. However, diversity of the mango landraces is largely unknown. We sought helps from local botanists and mango breeders, but still didn’t get much information. But together with Mr. Zhangguang Ni from Yunnan Province and Guodi Huang from Guangxi Province, we collected 9 additional landraces from remote areas in South China. We generated resequencing data for the germplasms newly collected, and also resequenced 18 additional cultivated varieties. Based on the analyses with bigger sample size, we observed that, despite forming independent clades, the landraces are close to the Southeast Asia germplasms. The mango germplasms are split into two major groups, coinciding with the proposed two centers of domestication. Also, we observed allelic admixture in the genomes of some landraces. Based on the updated results, we rewrote the “Mango germplasm diversity” section in Results, and revised Abstract and Discussion.*

**Second round of review**

**Reviewer 1**

The previous comments were addressed. no further comments.
